# Overexpression in *Plasmodium falciparum* of an intrinsically disordered protein segment of *Pf*UT impairs the parasite’s proteostasis and reduces its growth rate

**DOI:** 10.3389/fcimb.2025.1565814

**Published:** 2025-05-13

**Authors:** Yunuen Avalos-Padilla, Inés Bouzón-Arnáiz, Miriam Ramírez, Claudia Camarero-Hoyos, Marc Orozco-Quer, Elsa M. Arce, Diego Muñoz-Torrero, Xavier Fernàndez-Busquets

**Affiliations:** ^1^ Barcelona Institute for Global Health (ISGlobal), Hospital Clínic-Universitat de Barcelona, Barcelona, Spain; ^2^ Nanomalaria Group, Institute for Bioengineering of Catalonia (IBEC), The Barcelona Institute of Science and Technology, Barcelona, Spain; ^3^ Doctoral School of Biotechnology, Faculty of Pharmacy and Food Sciences, University of Barcelona, Barcelona, Spain; ^4^ Laboratory of Medicinal Chemistry, Faculty of Pharmacy and Food Sciences, University of Barcelona, Barcelona, Spain; ^5^ Institute of Biomedicine (IBUB), University of Barcelona, Barcelona, Spain; ^6^ Nanoscience and Nanotechnology Institute (IN2UB), University of Barcelona, Barcelona, Spain

**Keywords:** *Plasmodium falciparum*, E3 ubiquitin ligases, proteostasis disruption, protein aggregation, new antimalarial therapies

## Abstract

The proteome of *Plasmodium falciparum* exhibits a marked propensity for aggregation. This characteristic results from the parasite’s AT-rich genome, which encodes numerous proteins with long asparagine-rich stretches and low structural complexity, which lead to abundant intrinsically disordered regions. While this poses challenges for the parasite, the propensity for protein aggregation may also serve functional roles, such as stress adaptation, and could therefore be exploited by targeting it as a potential vulnerable spot in the pathogen. Here, we overexpressed an aggregation-prone segment of the *P. falciparum* ubiquitin transferase (*Pf*UTf), an E3 ubiquitin ligase protein that has been previously demonstrated to regulate the stability of parasite proteins involved in invasion, development and drug metabolism. Overexpression of *Pf*UTf in *P. falciparum* had evident phenotypic effects observed by transmission electron microscopy and confocal fluorescence microscopy, increased endogenous protein aggregation, disrupted proteostasis, and caused significant growth impairment in the parasite. Combined with dihydroartemisinin treatment, *Pf*UTf overexpression had a synergistic effect that further compromised the parasite´s viability, linking protein aggregation to proteasome dysfunction. Changes in the distribution of aggregation-prone proteins, shown by the altered subcellular fluorescent pattern of the new investigational aggregated protein dye and antiplasmodial compound YAT2150 in the overexpressing *P. falciparum* line, highlighted the critical balance between protein aggregation, stress responses, and parasite viability, suggesting proteostasis-targeting therapies as a good antimalarial strategy.

## 1 Introduction

Eukaryotic cells have evolved specialized mechanisms for rescuing misfolded proteins and selectively degrading mutant or damaged polypeptides to maintain protein quality and prevent cellular dysfunction (Tyedmers et al., 2010). When these systems fail, abnormal proteins can accumulate and form aggregates. This process of intracellular protein aggregation has gained interest in biology and medicine due to its significant implications for several neuropathological conditions in which certain peptides (e.g., amyloid-beta, α-synuclein, mutant huntingtin protein) form aggregates, impairing vital cellular processes (Ross and Poirier, 2004). Protein aggregation also occurs in response to cell stress, which can overwhelm the proteostasis system, leading to cellular dysfunction or apoptosis if unresolved (Grune et al., 2004; Ogen-Shtern et al., 2016; Hipp and Hartl, 2024). Nevertheless, protein aggregation also serves important biological purposes under certain circumstances, contributing to normal cellular function and adaptation. Examples include the transient aggregation of proteins during stress responses (Miller et al., 2015b; Saad et al., 2017), which acts as a protective strategy; aggregation as a storage mechanism, such as P-bodies in yeast (Buchan et al., 2008; Decker and Parker, 2012); cellular compartmentalization through the formation of membrane-less organelles (e.g., stress granules (Kearly et al., 2024) and aggresomes (Wang et al., 2009)); and functional amyloids, including curli fibers, which are responsible for biofilm formation in bacteria (Van Gerven et al., 2018).

The human malaria parasite species *Plasmodium falciparum*, known for being the most lethal of its kind, contains a notably high abundance of proteins with extended sequences of glutamine and asparagine repeats (Muralidharan et al., 2011; Muralidharan et al., 2012; Muralidharan and Goldberg, 2013; Pallarès et al., 2018). These proteins feature low complexity regions and tendency to form insoluble aggregates within cells (Halfmann et al., 2011). Moreover, studies have demonstrated that such protein aggregation occurs throughout all developmental stages of *Plasmodium* in both its vertebrate host and mosquito vector (Biosca et al., 2020). This includes gametocytes, the sexually committed parasite form present in the blood circulation, which are a preferred target of malaria transmission-blocking strategies (Chawla et al., 2021). Although the accumulation of protein deposits within a cell is commonly linked to disease pathology (Chiti and Dobson, 2017), in the case of malaria parasites, particularly *P. falciparum*, the presence of such protein aggregates is a notable feature with still not fully understood consequences for the viability of the pathogen. This distinctive phenotype offers a promising opportunity for developing novel therapeutic approaches, such as strategies that either enhance the accumulation of harmful aggregates or interfere with the parasite’s ability to handle them, thereby impairing its survival and replication. These targeted therapies could exploit *Plasmodium*’s reliance on protein homeostasis to gain a therapeutic advantage, offering a novel and potentially effective approach for combating malaria. Interestingly, substantial evidence suggests that protein aggregation may play a functional role in malaria parasites. For example, certain 4-aminoquinoline heterodimeric compounds, which exhibit antiplasmodial activity in the low micromolar range, have been identified as potent inhibitors of amyloid peptide aggregation (Espargaró et al., 2019). This finding raises the possibility that similar aggregation mechanisms might be crucial to the parasite’s biology or survival. Additionally, metabolites isolated from an *Eucalyptus* tree species with antiplasmodial activity were recently discovered to inhibit the aggregation of α-synuclein (Prebble et al., 2023). Remarkably, the active component of the commercial ProteoStat**^®^** reagent, used to detect intracellular protein aggregates *in vivo* (Biosca et al., 2020), is the bis(styrylpyridinium) salt YAT2150. This compound was found to kill *P. falciparum* in *in vitro* cultures with a half-inhibitory concentration (IC_50_) of ca. 90 nM and exhibits several properties desirable in the ideal antimalarial drug (Bouzón-Arnáiz et al., 2022). Its presumed mode of action is thought to involve the reduction of protein aggregation in live cells, as previously demonstrated by thioflavin T (ThT) assays performed in *P. falciparum* and *Leishmania infantum*-derived protein extracts following treatment with YAT2150 (Bouzón-Arnáiz et al., 2022; Román-Álamo et al., 2024).

To further investigate the potential of upsetting protein aggregation in *Plasmodium* as a new antimalarial strategy, we have engineered a parasite line to overexpress a segment of a highly aggregation-prone E3 ubiquitin-protein ligase (E3UL) termed *P. falciparum* ubiquitin transferase (Jankowska-Döllken et al., 2019) (*Pf*UT, 3893 amino acids, 460.4 kDa). This protein was previously identified in the *P. falciparum* protein pool that resisted solubilization in 0.1% sodium dodecyl sulfate (SDS), as detected by liquid chromatography with tandem mass spectrometry (LC-MS/MS) in two parasite samples prepared independently from late blood stages (Biosca et al., 2020) and early ring forms (Bouzón-Arnáiz et al., 2022). The hypothesis to be explored in this work is whether an induced increase of a highly abundant and aggregation-prone protein like *Pf*UT can further shift the parasite’s proteome towards an aggregation state surpassing its proteostasis control machinery, which might in turn lead to a decrease in its viability.

## 2 Results

### 2.1 Proteomic analysis of YAT2150-treated *P. falciparum* cultures

In a preliminary assay intended to explore the impact of YAT2150 treatment on protein aggregation levels in live parasites, we conducted a LC-MS/MS analysis focusing on individual proteins that exhibited resistance to solubilization in 0.1% SDS, a concentration that disrupts weak protein complexes while preserving the integrity of more stable aggregates (Kryndushkin et al., 2013). The analysis was carried out after subjecting a *P. falciparum* culture to a 20-h treatment with 0.2 µM YAT2150 (the compound’s IC_80_) and comparing it to an untreated control. The average aggregation propensity score for each dataset was calculated using the TAggregation predictor for Natural and Germline mOdeling (TANGO) algorithm, a computational tool widely used to predict protein aggregation propensity based on sequence properties. This tool identifies regions in a protein that are prone to forming β-sheet-rich aggregates by analyzing factors such as hydrophobicity, secondary structure, and backbone interactions (Fernandez-Escamilla et al., 2004). The mean TANGO score for proteins exclusively detected or showing greater abundance in the pellet of untreated samples and samples treated with YAT2150 was 8552 and 4259, respectively (**Supplementary Tables S1**, **S2**). This result was in agreement with the postulated activity of YAT2150 as inhibitor of protein aggregation in the malaria parasite, in particular of proteins with highest tendency to aggregate, as indicated by their significant removal from the 0.1% SDS-resistant pool when YAT2150 was present.

Gene Ontology (GO) term enrichment analysis was performed on the proteins exclusively found in the 0.1% SDS-resistant pellet of YAT2150-treated *P. falciparum* cultures (**Supplementary Table S2**) to characterize their functional and biological roles. While most of the identified genes remain unclassified, a significant proportion of the proteins were found to be involved in binding activities. Indeed, three proteins containing a PRESAN domain, present in *Plasmodium* helical interspersed subtelomeric (PHIST) proteins were identified. PRESAN is a targeting domain sufficient to localize *P. falciparum* proteins to the red blood cell periphery (Tarr et al., 2014), a phenomenon that has been suggested to play a key role in parasite survival through the modification of properties of both the erythrocyte cytoskeleton and plasma membrane. However, to elucidate the potential role of such protein export in the antiplasmodial mechanism of YAT2150 will be the object of future investigations.

On the other hand, some highly aggregation-prone proteins not found in the untreated control sample were enriched in the 0.1% SDS-insoluble fraction following treatment with YAT2150 (**Supplementary Table S2**). This result strongly suggests that although YAT2150 likely binds to unstructured protein regions —in most cases stabilizing (solubilizing) them—, in some proteins YAT2150 binding can lead to their destabilization and decreased solubility. One such protein identified only in the 0.1% SDS-resistant pellet of YAT2150-treated *P. falciparum* cultures was *Pf*UT, a homologous to the E6-associated protein (E6AP) C-terminus (HECT)-type E3 ubiquitin-protein ligase (C0H4K6) (Jankowska-Döllken et al., 2019). Previous LC-MS/MS analyses revealed *Pf*UT among the 23 proteins resistant to 0.1% SDS dissolution found in both early and late *P. falciparum* blood stages (Biosca et al., 2020; Bouzón-Arnáiz et al., 2022). Notably, *Pf*UT emerged as the most aggregation-prone protein within this protein dataset (Bouzón-Arnáiz et al., 2022). Moreover, studies carried by another group showed that overexpression of *Pf*UT hampered cell cycle progression and merozoite invasion (Jankowska-Döllken et al., 2019), possibly by *Pf*UT targeting for degradation, accidentally or in an untimely fashion, factors critical for invasion and cell cycle regulation. This led to a prolonged S/M phase, a slower overall replication rate, and a reduced merozoite invasion efficiency.

In the present study, we explored the phenotypic and biological effects of overexpressing a segment within the N-terminal disordered region of *Pf*UT in *P. falciparum*. This investigation was conducted within the broader context of studying protein aggregation in *P. falciparum*, whose understanding can significantly contribute to the development of novel efficient antimalarial strategies.

### 2.2 *P. falciparum* growth is not affected in ghost erythrocytes pre-loaded with the *Pf*UT aggregation-prone peptides KDLLF and KVVNI

Aggregation-prone regions are sufficient to promote the aggregation of complete proteins (Ventura et al., 2004), seeding significantly exacerbates aggregation reactions (Jarrett and Lansbury, 1993), and homologous seeding is far more efficient than heterologous seeding (Krebs et al., 2004). Although previous research successfully induced aggregation in live plant cells (Betti et al., 2016) and bacteria (Wu et al., 2021), similar efforts failed to elicit toxicity in human cell lines (Batlle et al., 2017) and *P. falciparum* (Bouzón-Arnáiz et al., 2022), probably due to the challenge of reaching a critical concentration of seeds required to maintain intracellular aggregation of the desired protein.

We first selected the KDLLF and KVVNI peptides, which are present in *Pf*UT and in other 10 and 9 *P. falciparum* proteins, respectively, to load ghost red blood cells (gRBCs) that were later used to establish parasite infection. These peptides were shown to form amorphous aggregates *in vitro* (Bouzón-Arnáiz et al., 2022), and when analyzed using WALTZ, a computational algorithm for predicting amyloidogenic sequences, they exhibited respective aggregation scores of 96.3 and 97.0. Unlike TANGO, which focuses on generic aggregation-prone regions, WALTZ specifically identifies sequences with high likelihood to form amyloid fibrils by analyzing structural and physicochemical properties, such as β-sheet formation and aggregation propensity (Maurer-Stroh et al., 2010). However, attempts to reduce the viability of *P. falciparum* parasites cultured in gRBCs loaded with KDLLF and KVVNI were unsuccessful [(Bouzón-Arnáiz et al., 2022) and **Supplementary Figure S1**]. Despite the peptides colocalized with a large fraction of parasitized gRBCs (pgRBCs), protein aggregation in the parasites was not significantly increased. This result might be explained by potential difficulties for exogenously added peptides to reach their target proteins. In confocal fluorescence microscopy assays done with KDLLF (**Supplementary Figure S2**), which was better incorporated in gRBCs than KVVNI, peptides inside gRBCs were observed to form aggregates not bounded by a membrane. However, this observation could not be unequivocally done in pgRBCs due to their complex membrane system. YAT2150 did not colocalize with peptides in gRBCs, but it did in pgRBCs, which likely represented the known YAT2150 interaction with endogenous protein aggregates in *P. falciparum* (Bouzón-Arnáiz et al., 2022), in regions where KDLLF also accumulated.

As an alternative strategy to seed protein aggregation in *Plasmodium*, we attempted the endogenous expression of self-aggregative peptides in the parasite. To achieve this, we used the p*HHI-cambsd* plasmid, which contains the *hsp86 P. falciparum* promoter that drives expression mainly in trophozoite stage parasites (Rug et al., 2004), and also encodes for a blasticidin S (BS) resistance gene (BS deaminase, BSD). Apart from conferring resistance to BS in *Plasmodium*, this plasmid enables the regulation of its copy number within the parasite by adjusting the amount of drug added to the culture; according to previous findings (Mamoun et al., 1999), at concentrations of 2 and 5 µg/mL BS, the parasite harbors 5 and 15 plasmid copies per cell, respectively.

A codon-optimized sequence encoding for either KDLLF or KVVNI was inserted between the promoter and terminator region of the *P. falciparum hsp86* gene (**Supplementary Figure S3**). However, after multiple attempts to transform the constructs into *Escherichia coli* nuclease-deficient strains, positive colonies were not obtained. This lack of success could be due to the aggregative nature of the selected peptides, which might have a toxic effect on the bacteria. Indeed, using the Basic Local Alignment Search Tool (BLAST, https://blast.ncbi.nlm.nih.gov/Blast.cgi, accessed on 01 October 2024) to analyze the *E. coli* proteome, we found that these peptides are present in 205 (KDLLF) and 169 (KVVNI) proteins from the bacterium. As described in previous reports (Wu et al., 2021), the peptides could be inducing aggregation of a large number of bacterial proteins, eventually producing a deleterious effect.

### 2.3 A *P. falciparum* line overexpressing a *Pf*UT segment shows growth delay and phenotypic alterations

**Figure 1:**
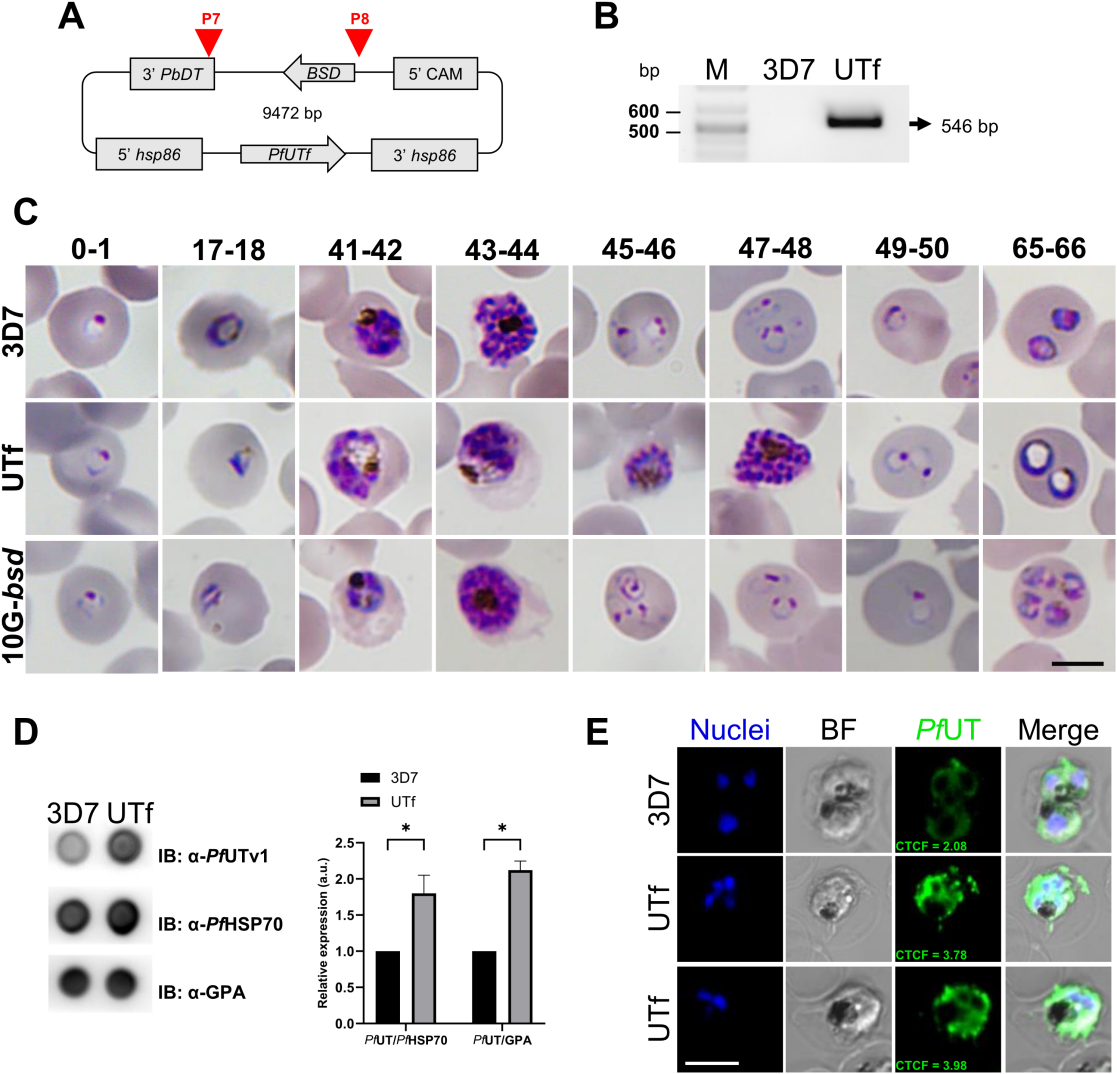
Overview of *Pf*UTf overexpression in *P. falciparum*. **(A)** Map of the p*HHI-cambsd_PfUTf* construct. Arrowheads indicate the oligonucleotides used in diagnostic PCR analysis (See **Supplementary Table S5**). **(B)** Diagnostic PCR showing the BSD cassette present in the stable UTf strain. Oligonucleotides P7 and P8 were used on total DNA prepared from 3D7 wild type and UTf strains. The arrow indicates the expected size of the amplified fragment. **(C)** Representative Giemsa-stained smears of *P. falciparum* strains 3D7, UTf, and 10G-*bsd* (the latter two maintained in the presence of 5 and 2.5 µg/mL BS, respectively). Images show different hours post infection (indicated at the top of the panel) after tight synchronization. Scale bar = 5 µm. **(D)** Representative dot blot assay and densitometry analysis demonstrating *Pf*UTf overexpression. Briefly, *P. falciparum* 3D7 or BS-treated UTf parasites were harvested, and 2 µg of lysates were probed with polyclonal anti-*Pf*UTv1 antibodies to detect protein expression relative to *Pf*HSP70 or glycophorin A (GPA) in parasite lines. n = 3; *p ≤ 0.05, Student’s *t* test; a.u.: arbitrary units. **(E)** Indirect immunofluorescence analysis of *Pf*UTf overexpression in cultures enriched in late-stage *P. falciparum* 3D7 or BS-treated UTf parasites. Rabbit polyclonal antibodies raised against *Pf*UTv1 were detected using anti-rabbit IgG Alexa 488-labeled secondary antibodies (green), and nuclei were counterstained with Hoechst 33342 (blue). Images show representative cells. Scale bar = 5 µm; BF, bright field; CTCF, corrected total cell fluorescence (a.u.).

Due to the toxic nature of the selected peptides for bacteria, we next explored the overexpression in *P. falciparum* of the region spanning from amino acid 76 to amino acid 518 of the *Pf*UT protein (*Pf*UTf, 52.3 kDa), which contains 10 aggregative and 8 low-complexity regions, according to WALTZ and Pfam algorithms, respectively (see **Supplementary Tables S3**, **S4**). For this, the coding sequence of the fragment was integrated into the p*HHI-cambsd* plasmid, resulting in the p*HHI-cambsd_PfUTf* construct (**Figure 1A**). Following the verification of the correct *PfUTf* insertion through sequencing, the construct was introduced into *P. falciparum* 3D7 parasites. Transfected parasites were maintained in Roswell Park Memorial Institute 1640 medium (RPMI) supplemented with 2 µg/mL BS, a concentration 5.7-fold higher than its IC_50_ in *P. falciparum* (Mamoun et al., 1999), until the establishment of the UTf stable line. The presence of the BSD cassette was confirmed in the obtained line by diagnostic PCR (**Figure 1B**). To evaluate the effect of *Pf*UTf overexpression, BS concentration was raised to 5 µg/mL in the culture medium and the growth of the parasite was monitored by Giemsa staining and light microscopy. As observed in **Figure 1C**, the transgenic parasites exhibited some defects particularly in late stages. These included the peripheral localization of hemozoin crystals (**Supplementary Figure S4A**) and an abundance of aberrant trophozoites in the transgenic culture, characterized by the presence of enlarged vesicles within the pathogen’s cytoplasm, observed in approximately 80% of the parasites (**Supplementary Figure S4B**). Moreover, transgenic parasites showed a prolonged intraerythrocytic developmental cycle (56.54 ± 4.26 h) compared to the parental 3D7 strain (45.65 ± 5.67 h) (**Supplementary Figure S4C**), and lower multiplication rates (1.98 ± 0.88), compared to 3D7 (7.70 ± 1.16). In our culture conditions, these two values were statistically different (*p* value = 0.0309). However, previous assays have shown that constant BS pressure in *P. falciparum* cultures downregulates the plasmodial surface anion channel activity, thereby negatively affecting nutrient uptake by the parasite and leading to slower growth rates (Hill et al., 2007; Mira-Martínez et al., 2013; Chan et al., 2020). To rule out the possibility that the observed effect was due to BS pressure on the culture, we included as a control for our experiments the *P. falciparum* 3D7A line 10G-*bsd* (10G-*bsd*), which carries the BS resistance episomal plasmid BsdR (Mira-Martínez et al., 2013), and is maintained in the presence of 2.5 µg/mL of BS. The transgenic UTf strain displayed a distinct phenotype from the 10G-*bsd* strain (**Figure 1C**), indicating that the observed effects were not due to the presence of BS. Moreover, the multiplication rate calculated for 10G-*bsd* was 5.78 ± 2.28, which is not statistically different from the value obtained for the 3D7 strain (*p* value = 0.3997).

The overexpression of *Pf*UTf was quantitated by dot blot assays using polyclonal antibodies raised against a smaller region of the protein, spanning from amino acid 177 to amino acid 535 (His-*Pf*UTv1, **Supplementary Figure S5A**). Enzyme-linked immunosorbent assays demonstrated that the raised antibodies exhibited specific recognition of the recombinant protein compared to preimmune serum (**Supplementary Figure S5B**). As expected, the overall signal corresponding to *Pf*UT increased significantly in the overexpressing *P. falciparum* line in comparison to the 3D7 strain (**Figure 1D**). The overexpression of *Pf*UTf in the transgenic strain was further confirmed by confocal fluorescence microscopy, particularly in trophozoites, a finding consistent with the activation pattern of the *hsp86* promoter at this stage of the parasite (**Figure 1E**, **Supplementary Figures S5C, D**). In these assays, the preimmune serum used as control did not elicit any detectable signal (**Supplementary Figure S5E**).

**Figure 2:**
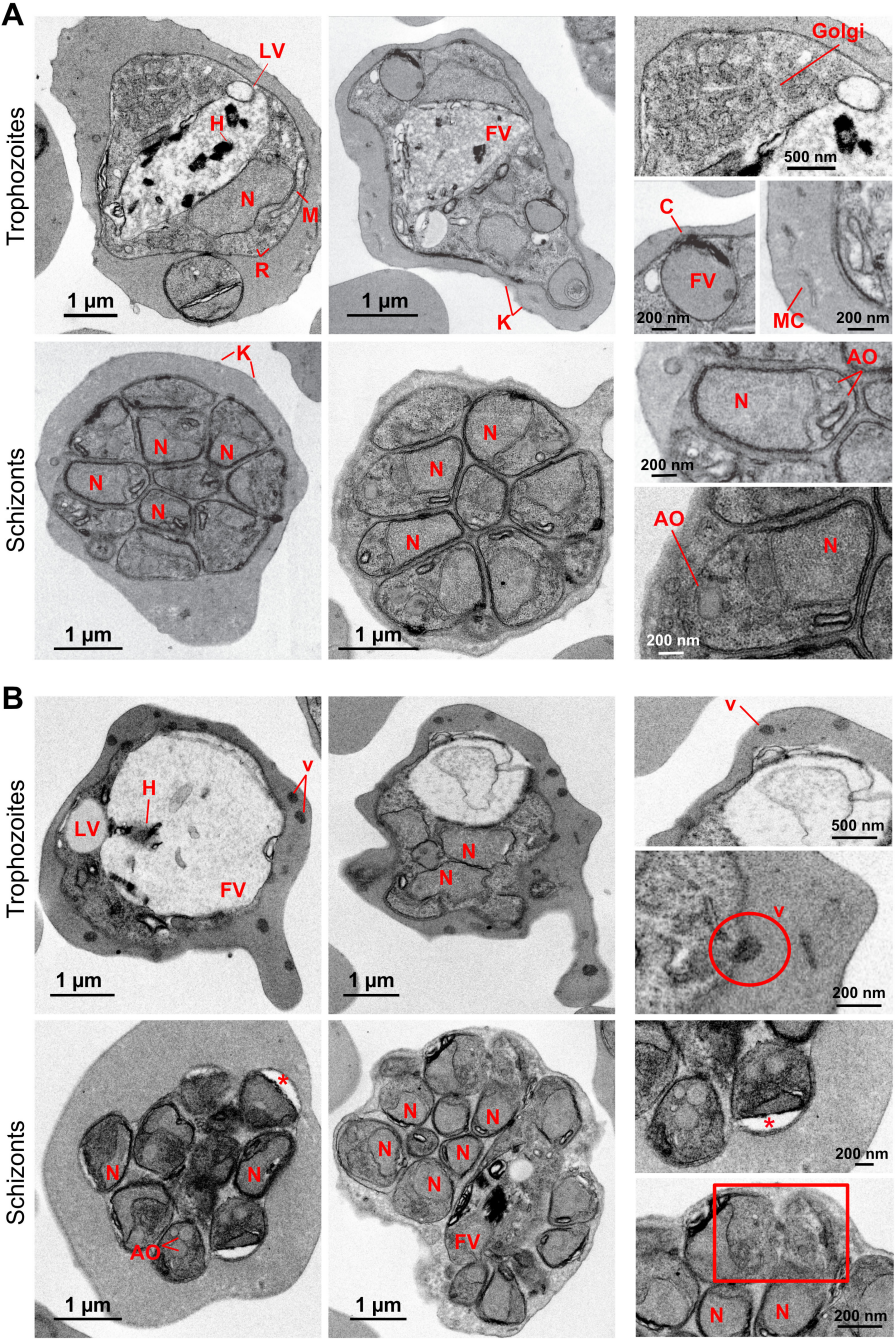
Transmission electron microscopy ultrastructural characterization of *Pf*UT overexpression in *P. falciparum*. **(A)** Micrographs of *P. falciparum* 3D7. In the cytoplasm of the parasite numerous ribosomes (R) and other structures including the nucleus (N), mitochondria (M), Golgi apparatus (Golgi), lipid vacuoles (LV) and food vacuoles (FV) containing hemozoin crystals (H) can be observed. Some food vacuoles were attached to a cytostome (C). K, knobs; MC, Maurer’s clefts; AO, apical organelles. **(B)** Micrographs of UTf parasites. Some electron-dense vesicles (v) are observed in the cytoplasm of trophozoites. The encircled area shows a nascent vesicle being expelled by the parasite to the host. In schizonts, less electron-dense areas are observed in some daughter cells (asterisks), but more segmented cells appear to have a normal morphology. In the rectangular boxed area, defective merozoites with disrupted membranes can be observed. The panels on the right side show zoomed-in images of the cells to provide a clearer view of the structures.

To further identify ultrastructural changes associated with *Pf*UTf overexpression that could explain the observed differences between 3D7 and UTf strains, we performed transmission electron microscopy (TEM) analysis (**Figure 2**). As shown in **Figure 1C**, Giemsa staining revealed that trophozoite and, to a lesser extent, schizont stages were affected in the overexpressing line. Therefore, samples were 70% Percoll synchronized in mature blood stages prior to fixation and preservation. Ultrastructural analysis allowed the identification of several organelles in the 3D7 line, including the nucleus, lipid vacuoles, mitochondria, Golgi apparatus, ribosomes, and the food vacuole, which contained a homogeneous electron-dense substance and hemozoin crystals (**Figure 2A**). Moreover, in 3D7 trophozoites, it was possible to detect the intense remodeling carried out by the parasite in the host erythrocyte, where sack-like cytosolic structures (Maurer´s clefts) and surface protrusions formed by electron-dense sub-membrane cups and the overlying RBC plasma membrane (knobs) were observed. Schizonts of the control line showed a normal morphology with different degrees of merozoite segmentation, where each daughter cell contained a nucleus and, in some cases, it was possible to observe other structures like apical organelles. In the case of the *Pf*UTf overexpressing line, a number of morphological changes were observed (**Figure 2B**), being more evident in the trophozoite stage. Food vacuoles were considerably larger than in the control sample, with limited hemozoin formation and loss of the electron-dense substance. This effect had previously been described (Pulcini et al., 2015) as a result of BS pressure, which leads to mutations in the *P. falciparum* chloroquine resistance transporter (*Pf*CRT) protein, located in the food vacuole (Pulcini et al., 2015). In normal conditions, *Pf*CRT transports peptides from the lumen of the parasite’s digestive vacuole into the cytosol, supplying essential amino acids for the metabolism of *Plasmodium* and preventing osmotic stress within the vacuole (Shafik et al., 2020). As its name suggests, mutations in *Pf*CRT are involved in chloroquine resistance (Fidock et al., 2000; Pulcini et al., 2015); however, the UTf line exhibited no significant differences in the IC_50_ value of chloroquine (15.54 ± 0.20 nM), compared to 3D7 (IC_50_ = 12.02 ± 0.24 nM) (**Supplementary Figure S6**). This suggests that the enlarged vacuole phenotype is likely due to the overexpression of *Pf*UTf and not a result of BS pressure in the culture.

Finally, dense vesicles were observed in the cytoplasm of the RBCs parasitized by the UTf line, where neither Maurer´s clefts nor knobs were detected. In schizonts, different degrees of segmentation were observed as in the control sample, but low electron-dense regions were detected in daughter cells (**Figure 2B**, asterisks). In some cases, the membrane of some merozoites appeared disrupted (**Figure 2B**, bottom right panel, square), which might lead to defective parasites and could explain the low invasion rates observed in culture conditions.

### 2.4 Protein aggregation is increased in parasites overexpressing *Pf*UTf

**Figure 3:**
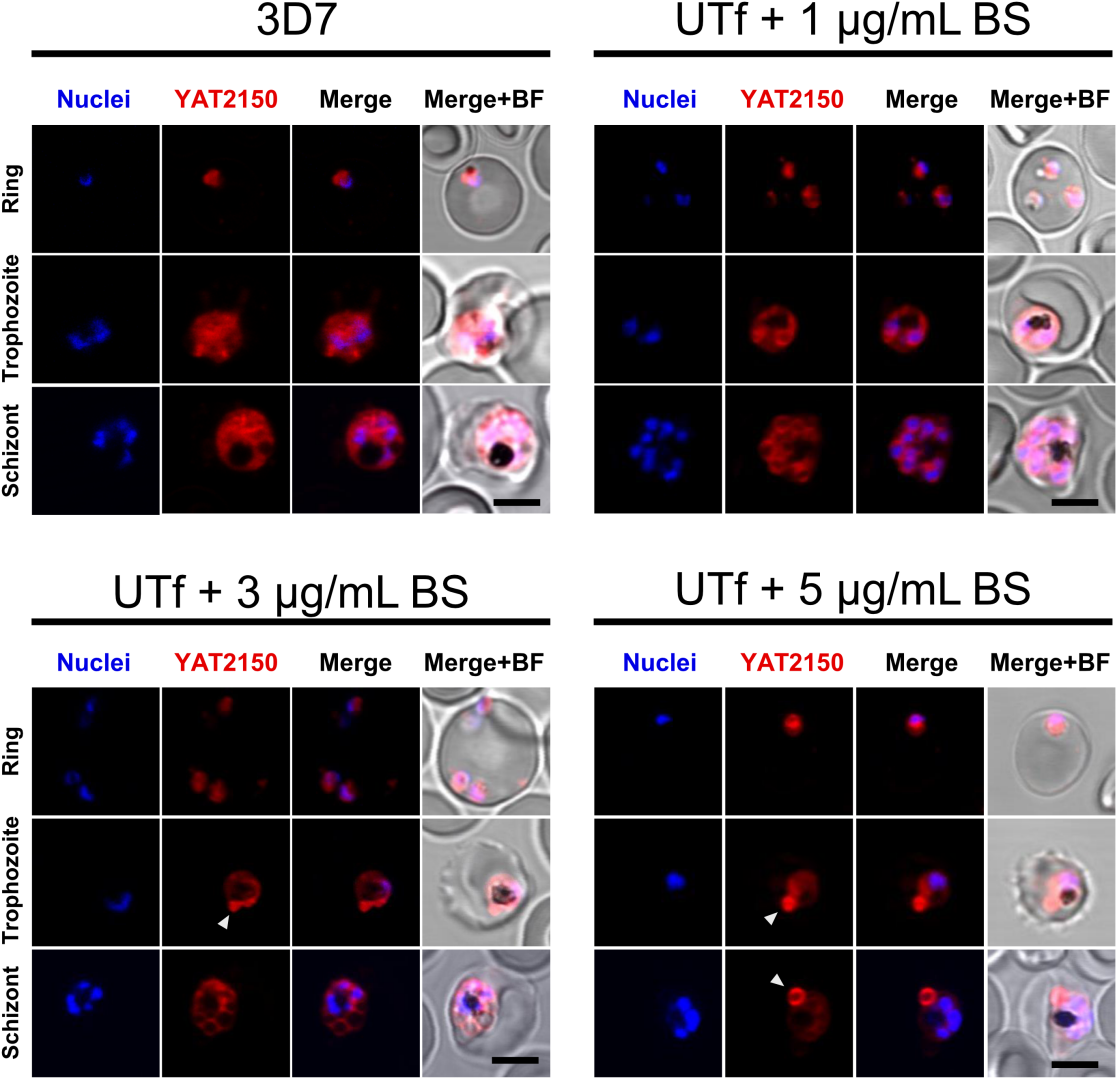
Confocal fluorescence microscopy analysis of YAT2150 distribution in *Pf*UTf-overexpressing parasites. Representative images show human erythrocytes infected with *P. falciparum* 3D7 or UTf parasites maintained at the different BS concentrations indicated over the corresponding panels. The cells were stained with 1 µM YAT2150 (red) to detect aggregated protein regions, and nuclei were counterstained with Hoechst 33342 (blue). The arrowheads indicate strongly fluorescent regions that may contain an accumulation of protein aggregates. Scale bar = 5 µm; BF, bright field.

Because the overexpressed region of *Pf*UT contained highly aggregative regions, potential alterations in protein aggregation in UTf parasites were explored by staining parasite cultures with the aggregated protein dye and antiplasmodial compound YAT2150 (Bouzón-Arnáiz et al., 2022), in the presence of different BS concentrations. According to confocal fluorescence microscopy analysis, the cytosolic distribution of the YAT2150-positive signal in UTf parasites was less homogenous than in the 3D7 strain (**Figure 3**). Additionally, certain confined regions with intense YAT2150 fluorescence were observed in UTf cultures maintained with 3 and 5 µg/mL BS, suggesting the accumulation of protein aggregates in those areas of the parasite (**Figure 3, arrowheads**).

**Figure 4:**
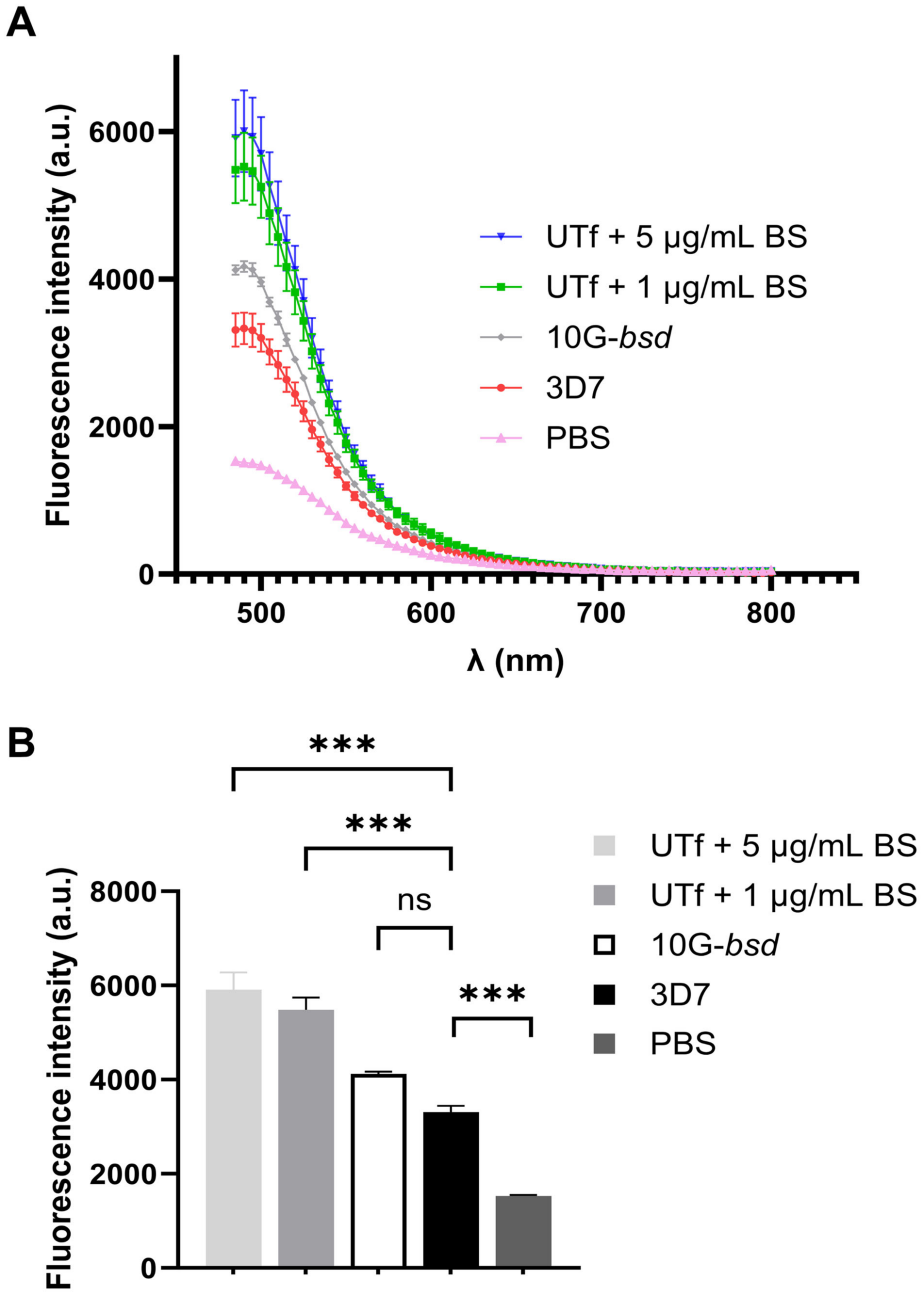
Effect on protein aggregation of *Pf*UTf overexpression. **(A)** ThT analysis in *P. falciparum* late-stage parasites. Briefly, total protein extracts from the different strains, normalized to have equal protein content, were incubated with ThT, and fluorescence was measured. **(B)** Means and standard deviations of the fluorescence intensities in panel A measured at the maximum emission wavelength of ThT (485 nm). n = 3; ***p ≤ 0.001 by one-way ANOVA; a.u., arbitrary units; PBS, phosphate-buffered saline; ns, not significant.

To further confirm changes in protein aggregation in UTf parasites, ThT was used to assess the extent of aggregation (Rusakov et al., 2024) in response to *Pf*UTf overexpression in live parasites. Our results demonstrated that the transgenic line exhibited a higher overall aggregation rate in comparison to 3D7, thereby confirming that our approach to induce protein aggregation in the parasite was successful (**Figure 4**). Moreover, we conducted dot blot assays using an amyloid structure-specific antibody and a detergent fractionation approach (Avalos-Padilla et al., 2021). Experimental data showed that amyloid-like structures increased in the 0.15% saponin fraction of UTf parasites, which corresponds to RBC cytosolic proteins and parasite-exported proteins (**Supplementary Figure S7**). Interestingly, a decrease in amyloid structures was observed in the radioimmunoprecipitation assay (RIPA) buffer fraction of transgenic parasites, containing cytoskeletal and aggregative components. The 1% Triton X-100 fraction, enriched in membranes and organelles from the parasites, showed no difference in amyloid content between 3D7 and UTf parasites.

### 2.5 UTf overexpression overloads *P. falciparum* proteostasis

**Figure 5:**
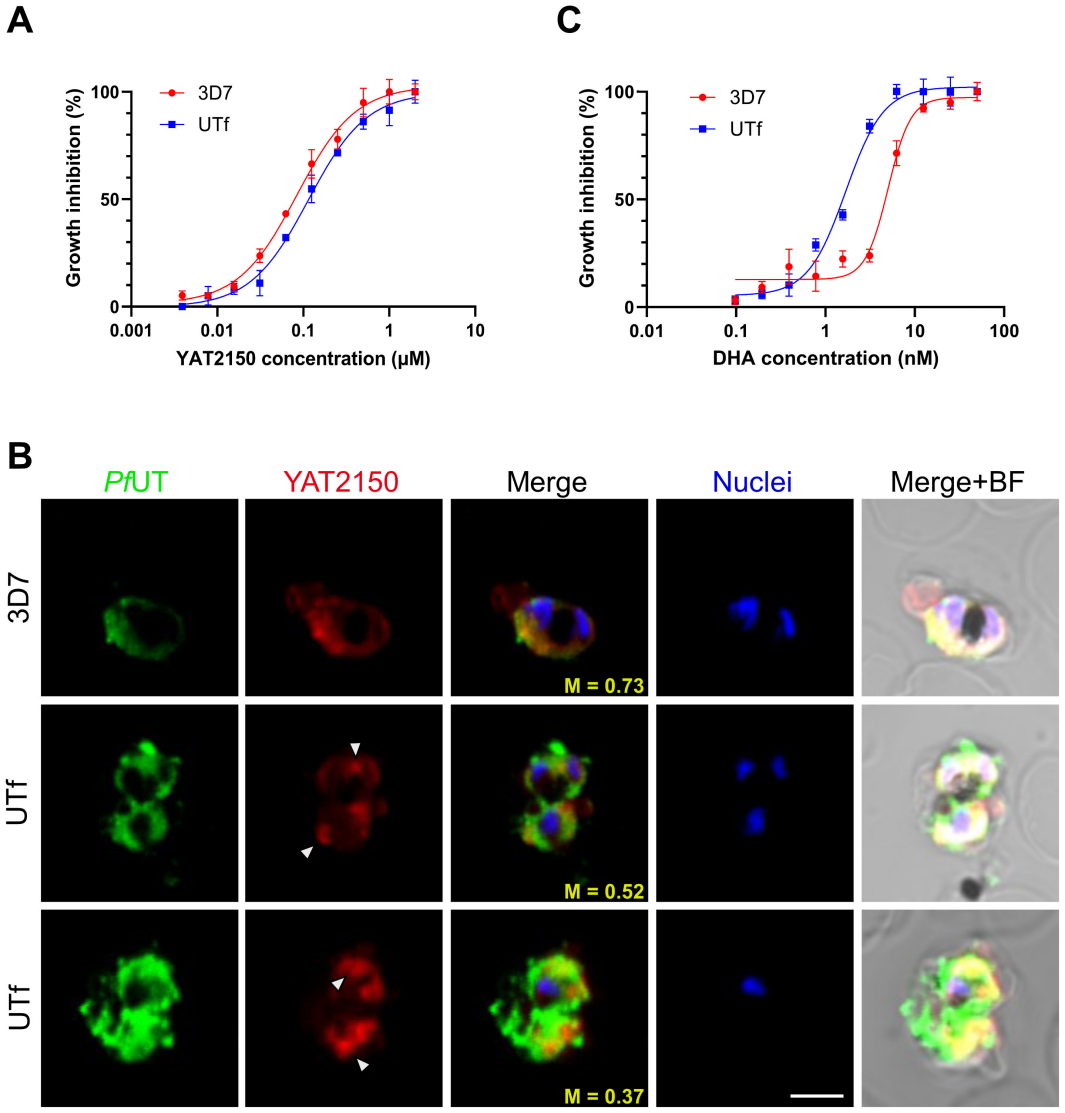
Analysis of the antiplasmodial activity of YAT2150 and DHA in the UTf line. **(A)** Growth inhibition assay of 3D7 and UTf parasites upon treatment with YAT2150. Data represents the mean ± SD of three independent replicates. **(B)** Representative confocal fluorescence microscopy images of human erythrocytes infected with *P. falciparum* 3D7 or UTf parasites. Parasites were labeled with YAT2150 (red), rabbit polyclonal antibodies raised against *Pf*UTv1 were detected using anti-rabbit IgG Alexa 488-labeled secondary antibodies (green), and nuclei were counterstained with Hoechst 33342 (blue). The arrowheads indicate strongly fluorescent regions that may contain an accumulation of protein aggregates. Scale bar = 5 µm; M: Manders’ coefficient; BF: bright field. **(C)** Growth inhibition assay of 3D7 and UTf parasites upon treatment with DHA. Data represents the mean ± SD of three independent replicates.

**Figure 6:**
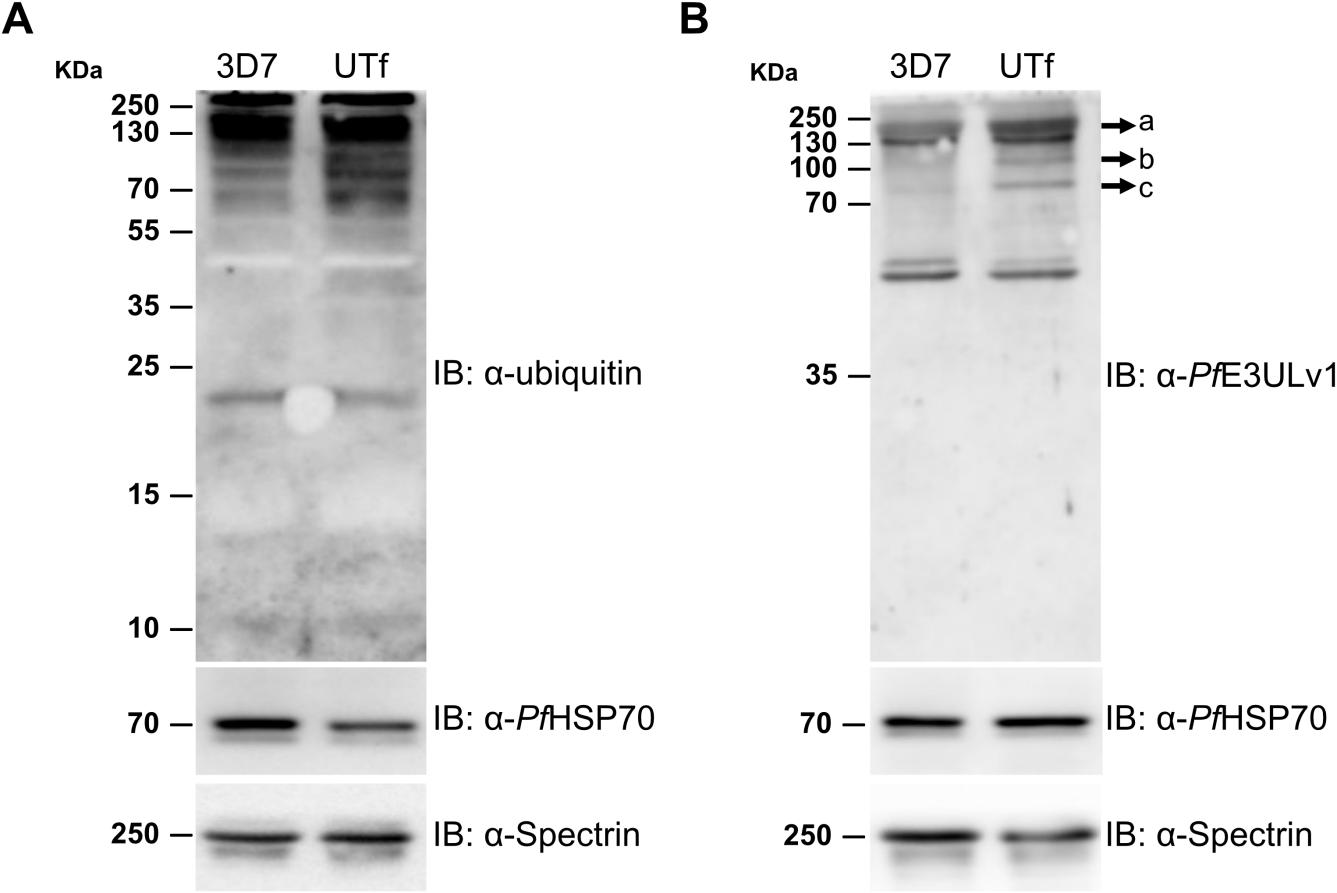
Analysis of the effect of *Pf*UT overexpression on protein aggregation. Representative Western blot assays for the detection in trophozoite stages of 3D7 and UTf lines of **(A)** ubiquitinated proteins and **(B)** *Pf*UT-enriched aggregates in a non-denaturing gel. Anti-*Pf*HSP70 and anti-spectrin antibodies were used as loading control shown at the bottom of each panel. IB, antibodies used in the corresponding immunoblots.

Previous studies indicated that the antiplasmodial mechanism of action of YAT2150 is most likely related to disturbing the aggregation-prone proteome of the parasite (Bouzón-Arnáiz et al., 2022). Once confirmed the increase in protein aggregation in the UTf strain, we proceeded to characterize the sensitivity of this line to YAT2150. The IC_50_ of YAT2150 for UTf parasites maintained in the presence of 5 µg/mL BS was 103.1 ± 13.9 nM, similar to that for the 3D7 strain (90.5 ± 6.1 nM, **Figure 5A**). The absence of significant differences (*p* value = 0.2239) between the two lines in the antiplasmodial activity of YAT2150 suggests that the antiparasitic mechanism of YAT2150 is not directly related to *Pf*UTf overexpression. Indeed, co-localization assays between YAT2150 signal and *Pf*UTv1 antibodies revealed that, while the 3D7 strain displays a high level of colocalization as measured by Manders’ coefficient, this value decreases in the UTf strain (**Figure 5B**). This suggests that the overexpressed fraction of *Pf*UTf is not directly targeted by YAT2150. Based on previous observations suggesting that dihydroartemisinin (DHA) is able to disrupt the function of the proteasome system (Bridgford et al., 2018), we explored the effect of DHA in the transgenic line. In this case, a synergistic effect of *Pf*UTf overexpression and DHA activity was observed (**Figure 5C**), where the IC_50_ values in the UTf and 3D7 lines were 1.7 ± 0.3 nM and 5.1 ± 0.7 nM, respectively (*p* value = 0.0015). This synergism potentially indicates that the proteostasis system in *P. falciparum* is further destabilized by *Pf*UTf overexpression, which enhances DHA-induced stress, leading to parasite death. To further explore a possible correlation between protein homeostasis and *Pf*UTf overexpression, we performed Western blot assays using protein extracts derived from either the 3D7 or the UTf lines to evaluate ubiquitinated proteins. As observed, ubiquitination levels were higher in the UTf line, suggesting that proteasome-mediated degradation is exacerbated, as it serves as the primary pathway for clearing misfolded and aggregated proteins (**Figure 6A**). Moreover, when detecting *Pf*UT with our generated antibodies in a non-denaturing gel (allowing visualization of aggregates), we observed three distinct bands that were more prominent in the UTf line (a, b and c in **Figure 6B**). This suggests that *Pf*UT-containing aggregates accumulate as a result of *Pf*UTf overexpression, which surpasses the proteasome machinery of the parasite.

## 3 Discussion

The proteome of *P. falciparum* is highly prone to aggregation (Biosca et al., 2020). Despite its relatively small genome (Gardner et al., 2002) and streamlined proteome, which suggest a compact, efficient biological background, the high propensity to protein aggregation in this organism may reflect a fine-tuned evolutionary trade-off between functionality and instability. This property may confer the parasite specific adaptive advantages such as aiding in protein protection or facilitating the formation of stress-related aggregates that help it survive in hostile environments, including oxidative stress in red blood cells and febrile episodes in the human host. In yeast, protein aggregation has been proved to be part of a protective mechanism. During oxidative and heat stress, damaged or misfolded proteins are either refolded by chaperones like Hsp70 and Hsp104 (Wetzel, 1994) or immobilized by sequestrases (Hill et al., 2017), like the *S. cerevisiae* proteins Btn2 and Hsp42 (Miller et al., 2015a; Carter et al., 2024). Despite no homologous of Btn2 and Hsp42 are found in the *P. falciparum* proteome, the parasite encodes a robust proteostasis protein network to handle the high burden of protein misfolding under the challenging conditions of the human host and vector environments. For instance, the chaperone Hsp110c plays a crucial role in maintaining proteostasis by efficiently preventing aggregation of the parasite’s asparagine repeat-rich proteome under febrile conditions (Muralidharan et al., 2011). Therefore, it is plausible that mechanisms like those observed in yeast are occurring in *Plasmodium*, but further experiments are required to test this hypothesis.

On the other hand, stress granules found in plants are cytoplasmic aggregates of proteins and mRNA that form in response to stress (Decker and Parker, 2012). This allows the organism to conserve energy and resources by selectively translating proteins essential for survival under adverse conditions. Previous LC-MS/MS analysis of highly aggregative proteins in live *P. falciparum* cells (Biosca et al., 2020) revealed an abundance of transcription factor (TF) apetala 2 (AP2) family members (Balaji et al., 2005), which mediate transcriptional regulation in response to stress conditions such as heat shock (Tintó-Font et al., 2021). The overrepresentation of *Pf*AP2s in the aggregative protein pool suggests a potential mechanism in the parasite to sense and respond to stress that could be dependent on intermolecular interactions such as those found in liquid-liquid phase separation-driven condensates and other forms of protein aggregation. Disrupting these dynamics with drugs like YAT2150 could significantly compromise the parasite’s viability.

In our study, a group in the pool of proteins not dissolving in 0.1% SDS after YAT2150 treatment belonged to gene products associated with catalytic activity (see **Supplementary Table S2**), among which E3UL *Pf*UT was detected. *P. falciparum* encodes 54 E3UL enzymes (Ponts et al., 2008), whose principal role is thought to be protein ubiquitination, an important post-translational modification, which has been shown to be essential for merozoite differentiation (Ponts et al., 2011) and parasite survival (Jain et al., 2017). Some examples of this family of proteins include CRL4 (cullin-RING E3 ligase 4) (Rizvi et al., 2024), involved in cell division, membrane integrity and drug resistance; *Pf*UT, which regulates the stability of proteins involved in invasion, development and drug metabolism (Sanchez et al., 2014; Jankowska-Döllken et al., 2019); and *Pf*RFUL, which modifies the roles of *Pf*MDR1 and *Pf*CRT, two proteins associated with drug resistance (Singh et al., 2023). Due to their high specificity for *P. falciparum* proteins (Ponts et al., 2011), E3ULs have been proposed as promising targets for new antimalarial drug development (Chung et al., 2012; Jain et al., 2017).

Our initial attempt to overexpress the peptides KDLLF and KVVNI found in *Pf*UT was unsuccessful. The inability to obtain positive colonies after transforming the peptide-carrying constructs into *E. coli* suggested that the codon-optimized sequences of the selected peptides might trigger a toxic effect in the bacteria. This toxicity is likely due to the aggregative properties of these peptides, which, when expressed, may induce the aggregation of multiple endogenous bacterial proteins as seen in other studies (Wu et al., 2021). Indeed, BLAST analysis of the *E. coli* proteome revealed that KDLLF and KVVNI are present in many of its proteins, indicating a high likelihood of cross-reactivity with them.

Given the challenges encountered with overexpressing small peptides, we selected a *Pf*UT section containing 10 aggregative and 8 low-complexity regions. This fragment is located at the N-terminus and clearly separated from the catalytic HECT E3 ligase domain found in the C-terminal 353 residues of the full-length protein (Jankowska-Döllken et al., 2019). Consequently, an increase in E3UL activity in the overexpressing line was not expected; instead, an exacerbation of protein aggregation was anticipated. However, Western blot assays revealed a higher level of ubiquitination in the UTf line compared to the 3D7 strain (**Figure 6A**), suggesting that the parasite’s quality control mechanisms are being activated to manage the potentially toxic increased protein aggregates in the overexpressing line. Moreover, analysis in a non-denaturing gel of *Pf*UT expression in the UTf line suggested the accumulation of *Pf*UT-containing aggregates (**Figure 6B**). These findings imply that the increased aggregation compromises the parasite’s protein degradation system, contributing to cellular stress and affecting *Plasmodium* viability. ThT assays further confirmed an overall increase in protein aggregation (**Figure 4**), which was accompanied by slower progression (**Supplementary Figure S4C**) and several morphological alterations in the parasite, including enlargement of the food vacuole (**Figure 1C**, **Figure 2**).

Since hemozoin is primarily formed in the food vacuole as a by-product of hemoglobin digestion (Sullivan et al., 1996), the abnormal enlargement of the vacuole may lead to the peripheral localization of hemozoin observed in the UTf line (**Supplementary Figure S4A**). Because the plasmid used for *Pf*UT overexpression is under the regulation of BS, this antibiotic was always present throughout our experiments. Previous studies have shown that BS pressure can induce numerous effects in the parasite, including slower growth rates (Mira-Martínez et al., 2013), and enlargement of the food vacuole due to mutations in the *Pf*CRT transporter (Pulcini et al., 2015). However, the analysis of a different *bsd*-carrying strain (10G-*bsd*), which showed a distinct phenotype from the UTf line, and the unchanged sensitivity to chloroquine of the transgenic strain relative to the 3D7 wild type (**Supplementary Figure S6**), indicated that the observed effects were due to *Pf*UTf overexpression and not to BS pressure. To further investigate the mechanism behind the enlargement of the digestive vacuole, future whole-genome sequencing could identify other mutations that might explain this phenotype.

Another characteristic of the UTf line was that the aggregated protein dye and antiplasmodial compound YAT2150 did not target the overexpressed *Pf*UTf protein, as suggested by (i) the lack of increased sensitivity to YAT2150 (**Figure 5A**), (ii) reduced co-localization in the UTf line when compared to 3D7 (**Figure 5B**), and (iii) the absence of a synergistic effect between YAT2150 and *Pf*UTf overexpression (**Figure 5A**). This could be due to structural differences between the aggregates formed by *Pf*UTf and those targeted by YAT2150, suggesting that the observed decrease in co-localization is not due to inaccessible binding sites, which might occur in an overexpressing line where aggregates can be densely packed or shielded, but rather to YAT2150 not binding *Pf*UTf aggregates. We have also observed that overexpression of *Pf*UTf exhibited a synergistic effect with DHA treatment (**Figure 5C**). This antimalarial drug is known to alter the proteasome, and *Pf*UTf overexpression could also be upsetting the correct function of this system. Indeed, YAT2150 distribution changed in the *Pf*UTf line, showing highly fluorescent, relatively small areas targeted by the compound (**Figure 5B**), indicating that most likely the distribution of other proteins targeted by YAT2150, and potentially aggregative, is also altered. Furthermore, DHA treatment has been shown to increase the number of released PI3P-containing vesicles (Bhattacharjee et al., 2018), which are key to artemisinin resistance in *P. falciparum* by spreading resistance intermediates that counteract drug-induced proteinopathy in *Plasmodium*-infected red blood cells. These vesicles, support proteostasis by promoting membrane curvature for macroautophagy, and their ability to reach multiple organelles explains the complexity of resistance mechanisms. This vesicle-based process could be also relevant to our system, as electron-dense vesicles were observed in TEM images of UTf parasites (**Figure 2B**). Dot-blot analysis further revealed that a substantial portion of amyloid-like proteins is exported to the red blood cell cytoplasm in the transgenic line, as demonstrated by their enrichment in the saponin fraction (**Supplementary Figure S7**). We hypothesize that the electron-dense vesicles observed by TEM may play a role in this process; however, this study is beyond the scope of the present work.

In conclusion, the overexpression of a highly aggregation-prone fragment of *Pf*UT produced a clear phenotypic effect in *P. falciparum*, resulting in parasites with significantly impaired growth rates. This overexpression increased the overall protein aggregation within the parasite, which in turn disrupted normal proteostasis. The synergistic effect of *Pf*UT overexpression and DHA treatment likely further compromised the proteasomal system, suggesting a link between protein aggregation and proteasome dysfunction. Notably, changes in the distribution of other aggregative proteins were observed, as evidenced by altered localization of YAT2150 (**Figure 3**). However, it is important to note that this observation is based on qualitative assessment rather than quantitative analysis, which could introduce subjectivity. The resolution limit of confocal microscopy, potential uneven staining, and differences in YAT2150 penetration or binding efficiency between samples could all contribute to this apparent heterogeneity. Future studies employing quantitative imaging approaches or complementary methods such as correlative light and electron microscopy, would be required to accurately compare protein distribution between the two strains.

Altogether, these findings highlight the fine balance between protein aggregation, cellular stress responses, and the parasite’s viability, pointing to potential therapeutic strategies targeting proteostasis pathways to combat *Plasmodium* infections.

## 4 Material and methods

Unless otherwise stated, reagents were purchased from Sigma-Aldrich Corporation (St. Louis, MO, USA), and solutions were made in bi-distilled H_2_O (MilliQ system, Millipore Corporation, Burlington, MA, USA).

### 4.1 LC-MS/MS analysis of aggregative proteins in *P. falciparum* cultures

*P. falciparum* tightly synchronized in ring stages were treated for 20 h with the IC_80_ of YAT2150 (0.2 µM) or left untreated for the same time under identical standard culturing conditions. Afterwards, proteins not solubilized in 0.1% SDS were extracted for each sample and subjected to LC-MS/MS as previously described (Biosca et al., 2020). Briefly, protein samples were digested with trypsin (Trypsin Gold, Promega Corporation, Madison, WI, USA) in a ProGestTM automatic digestor (Genomic Solutions, Inc., Ann Arbor, MI, USA) for 16 h at 37 °C. Mass spectrometry was performed using a NanoAcquity HPLC system (Waters Corporation, Milford, MA, USA) coupled to an Orbitrap Velos mass spectrometer (Thermo Fisher Scientific, Waltham, MA, USA). Peptides were separated in a C18 column (75 µm internal diameter, 25 cm length, 1.7 µm particle; NanoAcquity BEH column, Waters Corporation) with a gradient elution and analyzed in data-dependent mode, selecting the 15 most abundant peptides (≥500 counts) for fragmentation. Data were collected with Thermo Scientific Xcalibur software (v. 2.2) and analyzed using the SequestHT search engine in Proteome Discoverer (v. 1.4.1.14) against a database combining SwissProt human entries, *P. falciparum* (3D7) entries, and common contaminants. False discovery rate (FDR) estimation was performed to validate peptide-spectrum matches.

The relative abundance of each protein in both datasets was calculated according to the exponentially modified protein abundance index (emPAI) method (Ishihama et al., 2005). To compare abundance between treated and untreated cultures, log_2_ of the fold-change was calculated for each protein, being the fold-change the result of dividing the relative abundance in the YAT2150-treated dataset by that in the untreated dataset. Afterwards, proteins showing a log_2_ of the fold-change higher than +0.5 or lower than −0.5, or proteins exclusively detected in one of the two datasets, were analyzed using the TANGO algorithm (Fernandez-Escamilla et al., 2004) in order to check their aggregation propensity. The average of the aggregation propensity values (AGG parameter in TANGO) of each dataset was finally calculated.

### 4.2 Selection of the fragment inside *Pf*UT to be overexpressed in *P. falciparum*

The entire sequence of the C0H4K6 protein (E3 ubiquitin-protein ligase, *Pf*UT) was analyzed using the WALTZ algorithm (Maurer-Stroh et al., 2010) to identify short aggregative stretches. Additionally, a search was conducted on Pfam (Mistry et al., 2021) to identify low complexity regions (LCRs) (see **Supplementary Table S4**). According to these results, the selected region for overexpression spans from amino acid 76 to amino acid 518. Within this fragment, there are 10 aggregative peptides and 8 LCRs.

### 4.3 Generation of overexpressing strains

The plasmid used for overexpressing in *P. falciparum* the peptides KDLLF and KVVNI and the protein fragment *Pf*UT_76-518_ (*Pf*UTf) was derived from the p*HHI-cambsd-Dicre* construct (Llorà-Batlle et al., 2020), which features the *hsp86* promoter and terminator from *P. falciparum*. The removal of the *Dicre* cassette was accomplished using the *Eco*RI and *Age*I/*Pac*I restriction sites to excise the *Cre59* and *Cre60* genes, respectively, resulting in the formation of the p*HHI-cambsd* plasmid.

In the case of the peptides, cloning was attempted by annealing oligonucleotides encoding for either KDLLF (5’-ATGAAGGACTTATTATTCTAG-3’) or KVVNI (5’-ATGAAAGTTGTGAACATATAG-3’) into the *Age*I and *Pac*I sites of the p*HHI-cambsd* plasmid (for oligonucleotide sequences see **Supplementary Table S5**, P1-P4), using the In-Fusion system (Clontech, Takara Bio, Inc., Kusatsu, Japan), following the manufacturer´s protocol. The region encoding residues 76–518 of the HECT-type E3 ubiquitin ligase of *P. falciparum* (PF3D7_0704600) was PCR amplified using genomic DNA isolated from late-stage *P. falciparum* 3D7 parasites. Primers used for PCR amplification introduced unique *Age*I and *Pac*I sites in the sense and antisense strands (P5 and P6 in **Supplementary Table S5**), respectively, and a stop codon was also included in the antisense strand for the generation of a truncated protein. Subsequently, the annealed oligonucleotides or PCR product was cloned into the p*JET1.2/blunt* plasmid (Thermo Fisher Scientific) following the manufacturer´s instructions, and was later subcloned into the p*HHI-cambsd* plasmid to generate the p*HHI-cambsd_KDLLF*, p*HHI-cambsd_KVVNI* and p*HHI-cambsd_PfUTf* constructs. The presence of the inserts in the plasmids was further confirmed by restriction with *Eco*RI and Sanger sequencing. The generated plasmids were transformed into protease-deficient *E. coli* HST08 competent cells (Takara Bio, Inc.) to produce higher plasmid yields.

For transfection of 3D7 rings, 60 µg of p*HHI-cambsd_PfUTf* were precipitated, washed and resuspended in 30 mL of sterile 10 mM tris, pH 8.0, 1 mM EDTA (TE) buffer. Then, plasmids were diluted in 370 µL of Cytomix buffer (120 mM KCl, 0.15 mM CaCl_2_, 10 mM K_2_HPO_4_/KH_2_PO_4_, 2 mM EGTA, 5 mM MgCl_2_, 25 mM Hepes, pH 7.6) and transfected into parasites by electroporation using a Gene Pulser Xcell system (Bio-Rad Laboratories, Inc., Hercules, CA, USA), at 310 V, 950 µF of capacitance and without resistance. Electroporated parasites were carefully recovered and resuspended in RPMI supplemented with 2 µg/mL BS (Thermo Fisher Scientific) until obtaining a stable line. The presence of the BSD cassette was confirmed by diagnostic PCR using specific primers (P7 and P8 in **Supplementary Table S5**). After confirming that the plasmids were present in the selected lines, BS concentration was raised to 5 µg/mL to increase the number of episomal plasmid copies and, therefore, the amount of overexpressed protein.

The fitness of the transgenic line, compared to the parental 3D7 line, was evaluated by measuring the percentage of newly formed rings in synchronized cultures. Initial parasitemia was measured at approximately 18 hours post-infection (hpi), followed by ring parasitemia measurements at various time points during the period when most schizont bursting and reinvasion events occurred (44 to 62 hpi). The final measurement was taken at 74 hpi, when all viable schizonts had burst. Data points were calculated as the proportion of rings relative to the total number of rings at the end of the assay. The data was then fitted to a sigmoidal dose-response curve to determine the time required to produce 50% of the rings in each population (Rovira-Graells et al., 2012).

### 4.4 Culture maintenance

*P. falciparum* 3D7, 10G-*bsd* (Mira-Martínez et al., 2013) (kindly donated by Dr. Alfred Cortés) and UTf parasites were cultured under standard conditions (2% O_2_, 5.5% CO_2_, and 92.5% N_2_ at 37 °C) in RPMI supplemented with 5 mg/mL Albumax II (Invitrogen, Waltham, MA, USA), and 2 mM L-glutamine. The addition of BS was essential to maintain a stable line expressing the plasmid in the case of *Pf*UTf-overexpressing parasites. 10G-*bsd* parasites were maintained in the presence of 2.5 µg/mL BS in the culture medium.

### 4.5 Generation of peptide-loaded gRBCs

The peptides KDLLF and KVVNI and their fluorescent versions (functionalized with a 5(6)-carboxyfluorescein at the N-terminus), were synthesized as previously described (Román-Álamo et al., 2024), and stored at –20 °C until use. To disaggregate them, 1 mg of each lyophilized peptide was dissolved in 1 mL of trifluoroacetic acid (TFA), mixed thoroughly, and then TFA was evaporated under an N_2_ stream. Next, 500 µL of 1,1,1,3,3,3-hexafluoro-2-propanol (HFIP; Honeywell Fluka-Thermo Fisher Scientific) were added, mixed well, and evaporated as before. This step was repeated twice to remove TFA completely. Finally, 500 µL of HFIP were added, and the appropriate volume was separated into aliquots adjusted to 20 µM peptide. The aliquots were left in a desiccator overnight to evaporate the solvent completely.

To load peptides into gRBCs, a previously described protocol was followed (Govindarajalu et al., 2019). Briefly, regular RBCs were washed twice with three times their pellet volume of ice-cold phosphate-buffered saline (PBS). Afterwards, the RBC pellet was resuspended in 500 µL of ice-cold lysis buffer (1 mM ATP, 5 mM K_2_HPO_4_) containing the disaggregated peptide at a concentration of 10 µM. After 1 h of incubation at 4 °C with constant stirring, gRBCs were spun down and ½ vol of resealing buffer (150 mM NaCl, 5 mM MgCl_2_, 1 mM ATP, 1 mM glutathione) was added, and the mixture was incubated for an additional hour at 37 °C with gentle stirring. Finally, the samples were washed four times with RPMI and adjusted to 50% hematocrit in RPMI, and the generated gRBCs were stored at 4 °C until use. Infection of gRBCs by *P. falciparum* to obtain pgRBCs was performed by establishing a new culture after a late-stage synchronization using a 70% Percoll purification.

The presence of fluorescent peptides inside gRBCs and pgRBCs was assessed by confocal fluorescence microscopy (see below) and by flow cytometry using a 5-laser LSRFortessa flow cytometer (BD Biosciences, San Jose, CA, USA), with the side- and forward-scatter on a logarithmic scale to determine the cell population, and 10,000 events acquired for each sample. Hoechst 33342 and fluorescein were detected by excitation with 350- and 488-nm lasers, and emission was collected with 450/50-BP and 525/50-BP filters, respectively.

### 4.6 Microscopic analysis of the effects of *Pf*UT overexpression

Briefly, tightly synchronized *P. falciparum* 3D7 and UTf parasites at 1 h post-infection were obtained through 70% Percoll centrifugation as described before (Lambros and Vanderberg, 1979), followed by either 5% sorbitol (3D7) or L-proline lysis (UTf). For the latter, seven pellet volumes of 280 mM L-proline (diluted in distilled water) were added and the resuspended cells incubated for 7 min at 37 °C (Mira-Martínez et al., 2013), when they were thoroughly rinsed and placed back in RPMI for subsequent studies. Synchronized parasites were monitored during 66 h by Giemsa-stained blood smears. Images were taken with an Olympus IX51 microscope equipped with a cooled CCD Digital Camera (Olympus, Tokyo, Japan).

### 4.7 Generation of polyclonal antibodies

Due to the highly aggregation-prone nature of *Pf*UT, a shorter version of the protein was expressed for antibody generation. The region encoding residues 177–535 of the *P. falciparum* HECT-type E3 ubiquitin ligase (PF3D7_0704600) was PCR amplified using genomic DNA isolated from late-stage *P. falciparum* 3D7 parasites. Primers used for PCR amplification are listed in **Supplementary Table S5** (P9 and P10). Then, the amplified gene was cloned using the In-Fusion system into the *Bam*HI and *Sal*I sites of the p*Cold TF* vector (Takara Bio, Inc.), which introduces a N-terminal 6×histidine tag and expresses trigger factor (TF) chaperone as a soluble fusion tag. The resulting His(6×)-TF-UT(177-535) protein (hereafter referred to as His-*Pf*UTv1) was expressed in *E. coli* C41 (DE3) cells for 24 h at 16 °C in Terrific Broth medium (24 g/L yeast extract, 20 g/L tryptone, 0.4% glycerol, 0.017 mM KH_2_PO_4_, 0.072 mM K_2_HPO_4_) under stirring. Afterwards, cells were lysed by sonication in His-binding buffer (40 mM Na_3_PO_4_, pH 7.4, 500 mM NaCl, 30 mM imidazole, 0.2 mg/mL lysozyme, 20 µg/mL DNAse I, 1 mM MgCl_2_ and 1 mM Pefabloc^®^ SC). Lysates were applied into HisTrap FF columns (Cytiva Life Sciences, Marlborough, MA, USA) previously equilibrated with binding buffer. His-*Pf*UTv1 was eluted using the His-binding buffer at increasing concentrations of imidazole until reaching 500 mM. The purity of the eluted protein was confirmed by SDS-polyacrylamide gel electrophoresis (SDS-PAGE) followed by Coomassie Blue staining. The protein was finally quantified and snap frozen in liquid nitrogen until use.

For antibody generation, 400 µg of His-*Pf*UTv1 were emulsified in complete Freund’s adjuvant (1:1) and inoculated into male *New Zealand* rabbits. Three additional doses of 200 µg each were injected at 21-day intervals and the animals were finally bled 90 days after first immunization. Polyclonal IgGs against His-*Pf*UTv1 (anti-*Pf*UTv1) were purified using protein A sepharose affinity chromatography. Pre-immune serum was collected before immunization for control experiments.

The antibody titer was determined using a capture enzyme-linked immunosorbent assay (ELISA) by adsorbing 20 ng/well of His-*Pf*UTv1 onto the plates. After incubation with the raised antibodies, horseradish peroxidase (HRP)-conjugated secondary antibodies were added, followed by the 3,3’,5,5’-tetramethylbenzidine substrate. The reaction was stopped with an acidic solution, resulting in a yellow color. Absorbance was measured at 450 nm using a Victor3 plate reader (PerkinElmer, Inc., Waltham, MA, USA), with higher absorbance values indicating stronger antibody binding.

### 4.8 Protein extraction, Western blots and dot blots

Erythrocytes parasitized by the *P. falciparum* 3D7 or UTf strains were washed with PBS supplemented with 1× cOmplete protease inhibitor cocktail (Hoffmann-La Roche, Basel, Switzerland). Subsequently, pellets were suspended in 6× volumes of 0.15% saponin for 10 min at 4 °C and centrifuged (10,000× g, 15 min, 4 °C). The remaining pellet was washed with PBS-cOmplete and incubated with RIPA buffer (40 mM tris-HCl, pH 7.4, 150 mM NaCl, 2 mM EDTA, 10% glycerol, 1% Triton X-100, 0.5% sodium deoxycholate, 0.2% SDS and 1× cOmplete protease inhibitor cocktail) under continuous stirring at 4 °C. Extracts were then sonicated for 2 s at 40% amplitude and incubated on ice for 15 min. Finally, extracts were centrifuged (20,000× g, 15 min, 4 °C) to eliminate undissolved debris. For anti-ubiquitin assays, *N*-ethylmaleimide was included in all the steps.

In Western blot assays for the detection of ubiquitinated proteins, 30 µg of protein extracts from *P. falciparum* 3D7 and UTf parasites were resolved by SDS-PAGE using 12% bis-tris acrylamide (Bio-Rad Laboratories). To detect *Pf*UT-enriched aggregates, protein extracts were run under native conditions (without SDS), loaded onto a 5% stacking gel and a 10% separating gel, and subjected to PAGE under non-reducing conditions. After electrophoresis, proteins were transferred onto nitrocellulose membranes (Cytiva Life Sciences), which were then blocked overnight with 5% skim milk dissolved in tris-buffered saline (TBS: 50 mM tris-HCl, pH 7.6, 150 mM NaCl) containing 0.5% Tween 20 (TBS-T). Following blocking, membranes were washed 3× with TBS-T and incubated overnight at 4 °C with either rabbit anti-ubiquitin antibodies (Cell Signaling Technology, Inc., Danvers, MA, USA; 1:1000 dilution) or rabbit anti-*Pf*UTv1 antibodies (1:200), both diluted in TBS-T containing 3% skim milk (TBS-Tsm). Subsequently, the membranes were washed 5× with TBS-T, and incubated for 1 h with goat anti-rabbit IgG HRP-labeled secondary antibodies (Abcam, Cambridge, UK; 1:10,000) dissolved in TBS-Tsm. Detection was performed using the ECL Prime Western blotting detection reagent (Cytiva Life Sciences).

For the loading control, membranes were incubated for 3 h at room temperature with either rabbit anti-*Pf*HSP70 antibodies (StressMarq Biosciences, Inc., Victoria, Canada; 1:10,000) diluted in TBS-Tsm, or mouse monoclonal anti-spectrin antibodies (α and β, Sigma-Aldrich, Saint Louis, MO, USA; 1:10,000) diluted in TBS-T. Membranes probed with anti-*Pf*HSP70 antibodies were processed as the other antibodies. For membranes probed with anti-spectrin antibodies, three washes with TBS-T were performed, followed by incubation for 1 h with sheep anti-mouse IgG HRP-labeled secondary antibodies (Cytiva Life Sciences; 1:10,000) dissolved in TBS-Tsm, and detected as previously described.

For dot blot analysis, 2 µg of protein extracts from the tested conditions were deposited onto a nitrocellulose membrane, and blocked overnight as described above. Membranes were probed for 3 h at room temperature with either rabbit anti-*Pf*UTv1 (1:200), rabbit anti-*Pf*HSP70 (1:10,000), rabbit anti-glycophorin A (Acris Antibodies GmbH, Herford, Germany; 1:5,000), or rabbit anti-amyloid fibril (AB2286, Millipore Sigma, Burlington, MA, USA; 1:500) antibodies diluted in TBS-Tsm. Subsequently, membranes were washed with TBS-T and incubated for 1 h with goat anti-rabbit IgG HRP-labeled secondary antibodies (1:10,000) dissolved in TBS-Tsm, and developed with ECL Prime Western blotting detection reagent.

### 4.9 Confocal fluorescence microscopy

Blood smear preparations of *P. falciparum* parasites from the 3D7 and UTf strains (the latter maintained in the presence of 5 µg/mL BS) at late stages were air-dried and fixed with a 9:1 mixture of acetone:methanol for 2 min at room temperature. The indirect immunofluorescence detection continued with a 1-h incubation at room temperature of the fixed parasites with anti-*Pf*UTv1 antibodies diluted 1:500 with 0.75% bovine serum albumin. Subsequently, smears were washed with PBS and incubated for 30 min with Alexa 488-labeled goat anti-rabbit IgG antibodies (1:500), and nuclei were counterstained with 5 µg/mL Hoechst 33342 (λex/em: 350/461 nm). For YAT2150 staining (λex/em: 500/610 nm), live or fixed *P. falciparum* parasites were incubated with 1 µM of the compound and 5 μg/mL of Hoechst 33342 for 30 min. Afterwards, samples were rinsed and placed in an 8-well chamber slide (ibidi GmbH, Gräfelfing, Germany).

For the observation of pgRBCs and gRBCs loaded with fluorescein-tagged peptides, samples were incubated with 2 µg/mL Hoechst 33342 and 10 µM YAT2150 for 20 min. Rhodamine-labeled wheat germ agglutinin (WGA) was used for membrane staining at a concentration of 2.5 µg/mL and incubated for 1 h at 37 °C. Afterwards, samples were washed, diluted 1:20 in PBS and placed in an 8-well chamber slide.

All observations were performed with a Leica TCS SP5 confocal microscope (Leica Microsystems, Wetzlar, Germany) equipped with a 63× objective of 1.4 NA. Hoechst 33342 was excited with a diode laser at 405 nm, Alexa 488 with the 488 nm line of an argon laser, and YAT2150 and rhodamine with a diode-pumped solid-state laser at 561 nm. Fluorescence emission for individual acquisition was collected in the ranges of 415–460 nm for Hoechst 33342, 501–570 nm for Alexa 488, 490–590 nm for YAT2150, and 570–655 nm for rhodamine. In the case of co-localization studies, the ranges 501–568 nm for Alexa 488, and 605–707 nm for YAT2150 were used. To quantify Manders’ overlap coefficient (Manders et al., 1993), images were analyzed using the Just Another Colocalization Plugin (JACoP) in the Fiji software. Corrected total cell fluorescence (CTCF) was calculated using the following formula:


CTCF = Integrated density – (Area of selected cell × Mean fluorescence of background readings),

using the values obtained with the Fiji software.

### 4.10 Transmission electron microscopy

*P. falciparum* 3D7 and UTf parasites cultured in the presence of 5 µg/mL BS were 70% Percoll-synchronized at late stages, and cells were fixed with 2.5% glutaraldehyde and 2% paraformaldehyde in 0.1 M sodium phosphate buffer, pH 7.4 (PB) (Karnovsky, 1965), and incubated at 4 °C during 30 min in a shaker. After centrifugation at 2,500 rpm for 5 min, the samples were washed (5 × 10 min, 4 °C) with PB. Then, a solution of 1% osmium tetroxide and 0.8% potassium ferrocyanide in PB was added to the sample and incubated for 1.5 h at 4 °C in the dark, and washed 4 times for 10 min with double distilled water at 4 °C to eliminate excess of osmium. After dehydrating the sample with increasing concentrations of acetone, infiltration into Spurr resin was performed followed by polymerization during 3 days in a stove at 60 °C. Ultrathin 60-nm sections of the resin stub were cut using a Leica UC7 ultramicrotome (Leica Microsystems). Ultrathin sections were stained with 2% aqueous uranyl acetate for 10 min and with Reynolds lead-citrate staining solution for 5 min, and then analyzed at 80 kV in a JEOL JEM-1010 transmission electron microscope coupled with a Gatan Orius SC1000 (model 832) digital camera at the TEM-SEM Electron Microscopy Unit, Scientific and Technological Centers of the University of Barcelona (CCiTUB).

### 4.11 Quantitative analysis of protein aggregation in live *P. falciparum* cultures

*P. falciparum* parasites from the UTf strain, predominantly in late stages at 4% parasitemia and cultured in the presence of different BS concentrations (1 and 5 µg/mL), 10G-*bsd* parasites cultured in the presence of 2.5 µg/mL BS, and 3D7 parasites cultured without BS were used to extract total protein fractions. A 70% Percoll centrifugation was performed to isolate parasitized erythrocytes, and pellets containing late-stage parasites were resuspended in 50 µL of osmotic lysis buffer (4.5 mg/mL NaCl supplemented with cOmplete EDTA-free protease inhibitor cocktail). In all cases, a control consisting of a suspension of non-infected RBCs, maintaining the same cell concentration as in the purified cultures, was also included. Samples were incubated overnight at 4 °C under constant stirring to release intracellular components. Lysed samples were centrifuged (14,000× g, 5 min), and the protein content in the supernatant was quantified. Finally, 30 µg of protein from each sample was further diluted with PBS to a final volume of 100 µL and plated in a 96-well plate in triplicates. For pgRBC and gRBC analyses, 18 µg of protein per sample was processed similarly for the assay.

Thioflavin T (ThT) was added from a stock solution in PBS to obtain a final concentration of 25 µM, and fluorescence emission was collected from 470 to 800 nm using an excitation wavelength of 450 nm (Infinite Nano+ multimode microplate reader, Tecan Trading AG, Männedorf, Switzerland). A blank measurement of each sample was done before adding ThT.

### 4.12 *P. falciparum* growth inhibition assays

3D7 and UTf parasites were synchronized with 5% sorbitol and L-proline, respectively, to obtain a culture enriched in ring stages. After synchronization, parasites were further diluted to 1.5% parasitemia, and the hematocrit was adjusted to 2%. The culture was distributed in 96-well plates and treated with increasing concentrations of either YAT2150, DHA or chloroquine in triplicates. A negative control of untreated parasites and a positive control of parasites treated with a lethal dose of chloroquine was added to each plate. Parasites were incubated for either 48 h (for YAT2150-treated samples) or 72 h (for DHA-treated samples) in standard culturing conditions (2% O_2_, 5.5% CO_2_, and 92.5% N_2_ at 37 °C). After the incubation period, 0.1 µM Syto 11 (Thermo Fisher Scientific) was used to stain nuclei. Parasitemia was assessed by flow cytometry using a LSRFortessa flow cytometer set up with the 4 lasers, 20 parameters standard configuration. Syto 11 fluorescence signal was detected by exciting samples at 488 nm and collecting the emission with a 530/30 nm bandpass filter. Growth inhibition was calculated taking as reference values both the growth rate of the untreated culture and the growth rate of the culture treated with chloroquine. Growth inhibition data was transformed through sigmoidal fitting and used to determine the compound’s concentration required for the reduction of *P. falciparum* viability by 50% (IC_50_).

## Data Availability

The original contributions presented in the study are included in the article/**Supplementary Material**. Further inquiries can be directed to the corresponding authors.
