# Supplementary material for: Overexpression in Plasmodium falciparum of an intrinsically disordered protein segment of PfUT impairs the parasite’s proteostasis and reduces its growth rate
Source: Front Cell Infect Microbiol. 2025 May 13;15:1565814. doi: 10.3389/fcimb.2025.1565814 (PMC12106546; doi:10.3389/fcimb.2025.1565814)
Supplement: Supplementary file 1 [file DataSheet1.docx]

Supplementary Material

Overexpression in *Plasmodium falciparum* of an intrinsically disordered protein segment of *Pf*UT impairs the parasite’s proteostasis and reduces its growth rate

Yunuen Avalos-Padilla^1,2,*^, Inés Bouzón-Arnáiz^1,2^, Miriam Ramírez^1^, Claudia Camarero-Hoyos^1,2^, Marc Orozco-Quer^2^, Elsa M. Arce^3^, Diego Muñoz-Torrero^3,4^, and Xavier Fernàndez-Busquets^1,2,5,*^

^1^Barcelona Institute for Global Health (ISGlobal), Hospital Clínic-Universitat de Barcelona, Rosselló 149-153, 08036 Barcelona, Spain.

^2^Nanomalaria Group, Institute for Bioengineering of Catalonia (IBEC), The Barcelona Institute of Science and Technology, Baldiri Reixac 10-12, 08028 Barcelona, Spain.

^3^Laboratory of Medicinal Chemistry, Faculty of Pharmacy and Food Sciences, University of Barcelona, Av. Joan XXIII, 27-31, 08028, Barcelona, Spain.

^4^Institute of Biomedicine (IBUB), University of Barcelona, Av. Diagonal 643, 08028 Barcelona, Spain.

^5^Nanoscience and Nanotechnology Institute (IN2UB), University of Barcelona, Martí i Franquès 1, 08028, Barcelona, Spain

*** Correspondence:**Yunuen Avalos-Padilla
[yavalos@ibecbarcelona.eu](mailto:yavalos@ibecbarcelona.eu)

Xavier Fernàndez-Busquets

xfernandez@ibecbarcelona.eu

**Table S1. Proteins in the fraction resisting dissolution in 0.1% SDS more abundant or only identified in control untreated *P. falciparum* cultures.**

| **UNIPROT**  **Accession number** | **Protein name** | **Abundance untreated (%)** | **Abundance YAT2150 (%)** | **Abundance fold change YAT2150/**  **untreated** | **log2 fold change** | **TANGO score (AGG)** |
| --- | --- | --- | --- | --- | --- | --- |
| C6KSV0 | Histone H3 | 0.415 | 0.263 | 0.633 | -0.659 | 128.019 |
| Q8I5S6 | Eukaryotic translation initiation factor 3 subunit A | 0.215 | 0.044 | 0.204 | -2.297 | 3999.370 |
| Q8IDR9 | 40S ribosomal protein S6 | 0.159 | 0.109 | 0.689 | -0.537 | 186.658 |
| O97320 | Histone H2A | 0.108 | 0.000 |  |  | 567.990 |
| Q8I431 | 60S ribosomal protein L4 | 0.077 | 0.038 | 0.499 | -1.004 | 768.996 |
| Q8I1X4 | Uncharacterized protein | 0.061 | 0.000 |  |  | 1900.830 |
| Q8ILB6 | Mitochondrial acidic protein MAM33, putative | 0.061 | 0.000 |  |  | 379.052 |
| O97331 | Uncharacterized protein | 0.056 | 0.000 |  |  | 2538.760 |
| Q8I5K4 | Heterochromatin protein 1 | 0.056 | 0.000 |  |  | 1071.690 |
| Q8IKS4 | Hemolysin III | 0.056 | 0.000 |  |  | 9601.140 |
| C6KSR6 | VFT protein | 0.051 | 0.000 |  |  | 2154.700 |
| O77389 | Formate-nitrite transporter | 0.051 | 0.000 |  |  | 8827.230 |
| O96263 | rRNA methyltransferase, putative | 0.041 | 0.000 |  |  | 3026.540 |
| Q8I3T4 | FACT complex subunit | 0.041 | 0.000 |  |  | 6634.340 |
| C6KT85 | GAS8-like protein, putative | 0.036 | 0.000 |  |  | 178.613 |
| C6S3B4 | Sugar phosphate phosphatase | 0.036 | 0.000 |  |  | 3084.220 |
| Q25799 | FeS cluster assembly protein SufB | 0.031 | 0.000 |  |  | 5698.050 |
| O96204 | Uncharacterized protein | 0.026 | 0.011 | 0.427 | -1.226 | 50971.100 |
| Q8I2A1 | Parasite-infected erythrocyte surface protein | 0.026 | 0.000 |  |  | 5347.750 |
| Q8II43 | CCT-alpha | 0.026 | 0.000 |  |  | 1551.760 |
| Q8IJD0 | Peroxiredoxin | 0.023 | 0.008 | 0.356 | -1.490 | 1373.350 |
| C6KST7 | Nucleoporin NUP637, putative | 0.020 | 0.005 | 0.267 | -1.905 | 43642.400 |
| Q8IKF6 | Kelch domain-containing protein, putative | 0.020 | 0.011 | 0.534 | -0.905 | 10775.000 |
| Q8IB24 | Heat shock protein 70 | 0.020 | 0.000 |  |  | 1344.780 |
| Q8ILI9 | DNA mismatch repair protein MSH2, putative | 0.020 | 0.000 |  |  | 4993.300 |
| Q8IJI4 | PHAX domain-containing protein, putative | 0.019 | 0.010 | 0.563 | -0.828 | 1912.720 |
| C0H4V4 | Rhoptry neck protein 5 | 0.015 | 0.000 |  |  | 8939.640 |
| C6KSR8 | Translation initiation factor IF-2, putative | 0.015 | 0.000 |  |  | 5168.920 |
| O77391 | CS domain-containing protein | 0.015 | 0.000 |  |  | 7398.230 |
| Q8I2K5 | Elongation factor Tu, putative | 0.015 | 0.000 |  |  | 7398.230 |
| Q8IEL1 | Uncharacterized protein | 0.015 | 0.000 |  |  | 5326.810 |
| Q8IIE1 | Uncharacterized protein | 0.015 | 0.000 |  |  | 3939.160 |
| Q8IJ92 | SAE2 domain-containing protein | 0.015 | 0.000 |  |  | 612.872 |
| Q8IKK6 | ATPase_AAA_core domain-containing protein | 0.015 | 0.000 |  |  | 4937.530 |
| Q8ILZ3 | CTP synthase | 0.015 | 0.000 |  |  | 3301.280 |
| C6KT64 | Leucyl-tRNA synthetase | 0.010 | 0.000 |  |  | 6847.100 |
| C6KT73 | ATP-dependent RNA helicase SUV3, putative | 0.010 | 0.000 |  |  | 5922.190 |
| Q8I1X5 | Pre-mRNA-processing-splicing factor 8, putative | 0.010 | 0.000 |  |  | 12234.400 |
| Q8I2B4 | Erythrocyte binding antigen-181 | 0.010 | 0.000 |  |  | 3330.690 |
| Q8II67 | Phosphatidylinositol-4-phosphate 5-kinase, putative | 0.010 | 0.000 |  |  | 6054.350 |
| Q8IJS7 | PRE-binding protein | 0.010 | 0.000 |  |  | 1646.590 |
| Q8ILD1 | Uncharacterized protein | 0.010 | 0.000 |  |  | 8470.090 |
| O77375 | DNA-directed RNA polymerase subunit | 0.006 | 0.001 | 0.203 | -2.298 | 3792.550 |
| Q8I3Q8 | Mediator of RNA polymerase II transcription subunit 17, putative | 0.006 | 0.005 | 0.814 | -0.298 | 4055.490 |
| C0H5A6 | Uncharacterized protein | 0.005 | 0.000 |  |  | 45074.100 |
| A0A143ZY53 | Erythrocyte membrane protein 1, PfEMP1 | 0.005 | 0.000 |  |  | 3817.240 |
| A0A144A5C9 | Uncharacterized protein | 0.005 | 0.000 |  |  | 11157.600 |
| C0H4B8 | Uncharacterized protein | 0.005 | 0.000 |  |  | 15256.900 |
| C6KT33 | Uncharacterized protein | 0.005 | 0.000 |  |  | 17678.200 |
| O77372 | Uncharacterized protein | 0.005 | 0.000 |  |  | 48311.300 |
| Q8I259 | Uncharacterized protein | 0.005 | 0.000 |  |  | 4594.030 |
| Q8I3X5 | CPSF_A domain-containing protein | 0.005 | 0.000 |  |  | 23011.800 |
| Q8I5T5 | Lysine-specific histone demethylase 1, putative | 0.005 | 0.000 |  |  | 10901.200 |
| Q8I639 | Erythrocyte membrane protein 1, PfEMP1 | 0.005 | 0.000 |  |  | 5869.700 |
| Q8IB09 | Uncharacterized protein | 0.005 | 0.000 |  |  | 1643.950 |
| Q8IBB0 | SET domain protein, putative | 0.005 | 0.000 |  |  | 5997.080 |
| Q8IBY8 | Uncharacterized protein | 0.005 | 0.000 |  |  | 6669.130 |
| Q8IDH3 | Intron-binding protein aquarius, putative | 0.005 | 0.000 |  |  | 19851.100 |
| Q8IEH7 | Clu domain-containing protein | 0.005 | 0.000 |  |  | 11865.900 |
| Q8IHU2 | Uncharacterized protein | 0.005 | 0.000 |  |  | 12998.700 |
| Q8IHY4 | Uncharacterized protein | 0.005 | 0.000 |  |  | 2635.290 |
| Q8II32 | Uncharacterized protein | 0.005 | 0.000 |  |  | 2558.320 |
| Q8IJL2 | Eukaryotic translation initiation factor subunit eIF2A, putative | 0.005 | 0.000 |  |  | 5468.340 |
| Q8IK03 | Uncharacterized protein | 0.005 | 0.000 |  |  | 8645.200 |
| Q8ILS4 | NOT family protein, putative | 0.005 | 0.000 |  |  | 21606.300 |
| C0H491 | NYN domain-containing protein, putative | 0.003 | 0.001 | 0.407 | -1.298 | 6809.110 |
|  |  |  |  | Mean TANGO score | | 8552 |

**Table S2. Proteins in the fraction resisting dissolution in 0.1% SDS more abundant or only identified in YAT2150-treated *P. falciparum* cultures.** Shadowed in gray, proteins associated with catalytic activity (GO:0003824).

| **UNIPROT**  **Accession number** | **Protein name** | **Abundance YAT2150 (%)** | **Abundance untreated (%)** | **Abundance fold change YAT2150/**  **untreated** | **log2 fold change** | **TANGO score (AGG)** |
| --- | --- | --- | --- | --- | --- | --- |
| Q8IBV7 | Histone H2B | 0.514 | 0.287 | 1.793 | 0.843 | 549.133 |
| C0H4A6 | Ribosomal protein L15 | 0.257 | 0.072 | 3.587 | 1.843 | 893.035 |
| O96258 | 40S ribosomal protein S26 | 0.148 | 0.000 |  |  | 24.470 |
| Q9TY99 | Knob-associated histidine-rich protein | 0.111 | 0.070 | 1.577 | 0.657 | 1063.920 |
| Q8I444 | Small ubiquitin-related modifier | 0.084 | 0.044 | 1.889 | 0.918 | 16.653 |
| Q8ILV2 | 60S ribosomal protein L10, putative | 0.071 | 0.000 |  |  | 129.837 |
| Q8IIV1 | Histone H2B | 0.068 | 0.000 |  |  | 429.057 |
| C0H4L6 | Uncharacterized protein | 0.055 | 0.015 | 3.561 | 1.832 | 1397.130 |
| O96283 | Stevor | 0.049 | 0.000 |  |  | 5486.540 |
| Q8IBY4 | 60S ribosomal protein L34 | 0.049 | 0.027 | 1.798 | 0.847 | 2.831 |
| O96190 | Uncharacterized protein | 0.038 | 0.000 |  |  | 5664.680 |
| Q8I0U6 | Ring-infected erythrocyte surface antigen | 0.034 | 0.007 | 4.718 | 2.238 | 3353.650 |
| Q8IIS9 | Polyadenylate-binding protein-interacting protein 1, putative | 0.027 | 0.015 | 1.781 | 0.832 | 3776.810 |
| O96127 | Translation-enhancing factor | 0.027 | 0.010 | 2.671 | 1.417 | 2092.670 |
| C0H513 | Serine/threonine protein kinase, FIKK family | 0.027 | 0.000 |  |  | 2693.650 |
| Q8I207 | PRESAN domain-containing protein | 0.027 | 0.000 |  |  | 2524.920 |
| Q8IE18 | RNA-binding protein, putative | 0.027 | 0.000 |  |  | 858.565 |
| Q8IJI0 | Serine/arginine-rich splicing factor 4 | 0.027 | 0.000 |  |  | 127.962 |
| Q8ILL5 | NTF2 domain-containing protein | 0.024 | 0.013 | 1.898 | 0.925 | 3109.060 |
| Q8IBR1 | Uncharacterized protein | 0.022 | 0.000 |  |  | 240.393 |
| Q8IAY2 | Uncharacterized protein | 0.022 | 0.000 |  |  | 976.773 |
| Q8IHN4 | Antigen 332, DBL-like protein | 0.016 | 0.005 | 3.205 | 1.680 | 3918.180 |
| O96232 | Acyl-CoA synthetase | 0.016 | 0.000 |  |  | 5033.950 |
| Q8I0V4 | Endoplasmin, putative | 0.016 | 0.000 |  |  | 3645.460 |
| Q8I355 | FHA domain protein, putative | 0.016 | 0.000 |  |  | 3033.770 |
| Q8I627 | Cytochrome C oxidase assembly protein COX19, putative | 0.015 | 0.000 |  |  | 470.279 |
| Q8ILP3 | Surface protein P113 | 0.014 | 0.004 | 3.254 | 1.702 | 5837.420 |
| Q8IAX8 | DNA/RNA-binding protein Alba 1 | 0.014 | 0.000 |  |  | 151.543 |
| Q8ILL2 | 60S ribosomal protein L7a | 0.013 | 0.000 |  |  | 575.864 |
| Q8IDR1 | Phosphoenolpyruvate carboxykinase (ATP) | 0.012 | 0.007 | 1.627 | 0.702 | 2751.640 |
| C6KT25 | L-lactate dehydrogenase | 0.012 | 0.000 |  |  | 2244.280 |
| Q8IJN0 | PRESAN domain-containing protein | 0.012 | 0.000 |  |  | 3067.050 |
| C0H4C6 | MMS19-like protein, putative | 0.011 | 0.005 | 2.137 | 1.095 | 34828.400 |
| C0H496 | Reticulocyte binding protein homologue 4 | 0.011 | 0.000 |  |  | 8696.130 |
| Q8II19 | GPI inositol-deacylase | 0.011 | 0.000 |  |  | 12181.900 |
| C0H4N3 | Transcription initiation factor IIE subunit alpha, putative | 0.008 | 0.000 |  |  | 1547.130 |
| C6KTB9 | Choline/ethanolaminephosphotransferase, putative | 0.008 | 0.000 |  |  | 13386.000 |
| Q8I2F2 | PRESAN domain-containing protein | 0.008 | 0.000 |  |  | 2431.850 |
| Q8I6T0 | Aminomethyltransferase, mitochondrial, putative | 0.008 | 0.000 |  |  | 1880.940 |
| Q76NM2 | Thrombospondin-related anonymous protein | 0.006 | 0.000 |  |  | 3181.700 |
| Q8IAV3 | Uncharacterized protein | 0.006 | 0.000 |  |  | 7238.820 |
| A0A144A0A3 | TBC domain-containing protein, putative | 0.005 | 0.000 |  |  | 12610.000 |
| C0H4K6 | E3 ubiquitin-protein ligase | 0.005 | 0.000 |  |  | 23374.400 |
| C6KSP3 | GYF domain-containing protein | 0.005 | 0.000 |  |  | 2306.290 |
| Q8IBF2 | EMP1-trafficking protein | 0.005 | 0.000 |  |  | 1798.390 |
| Q8IIQ7 | RNA-binding protein, putative | 0.005 | 0.003 | 1.627 | 0.702 | 462.569 |
| Q8IE65 | Uncharacterized protein | 0.002 | 0.001 | 1.627 | 0.702 | 8132.690 |
|  |  |  |  | Mean TANGO score | | 4259 |

| **Residues** | **Aggregative Sequence** | **WALTZ score** |
| --- | --- | --- |
| 2017-2028 | IFCNIENFYIYN | 97.80 |
| 2049-2059 | WNNNYNINNNN | 96.32 |
| 2118-2125 | NNNIYSLN | 96.32 |
| 2127-2134 | NNNIYSLN | 96.32 |
| 2138-2143 | NSVNYK | 92.64 |
| 2152-2157 | NNSLFM | 92.64 |
| 2198-2209 | SVSNYVDWVTYK | 96.66 |
| 2227-2239 | KYNYYNISYDLFK | 98.04 |
| 2299-2307 | FDNNINNSD | 97.32 |
| 2356-2364 | NELNFLFSQ | 96.80 |
| 2374-2383 | YLSNYLQIEM | 97.66 |
| 2386-2392 | ATELIFM | 96.89 |
| 2470-2479 | NVENIYKNYD | 96.66 |
| 2486-2497 | FYLLYNYMPIYN | 98.38 |
| 2540-2545 | GDNIYV | 98.33 |
| 2550-2559 | NLNNIESYYN | 97.06 |
| 2576-2590 | NMFNFLIYKFLYLLC | 96.52 |
| 2600-2608 | TFMNFYNNN | 96.10 |
| 2611-2619 | EYFKYNEYV | 94.54 |
| 2681-2690 | YLNEFNFIEN | 96.86 |
| 2711-2726 | NKVIYYLNNPLFIFSE | 95.84 |
| 2734-2745 | KIFYLFNFVIDI | 98.19 |
| 2751-2767 | FFELIYFGNYRGLYNYK | 96.81 |
| 2796-2801 | VCANYT | 95.21 |
| 2808-2821 | KLFNFLSDNNILSL | 95.51 |
| 2872-2877 | SLEFYN | 97.32 |
| 2906-2914 | NLNNFDFSL | 94.98 |
| 2968-2985 | VNNLINHFLFSMNSNIFQ | 96.80 |
| 3355-3362 | EVVIISSN | 97.99 |
| 3415-3420 | KIIIIN | 98.33 |
| 3434-3439 | NTVTIE | 92.64 |
| 3475-3480 | NENYIL | 96.99 |
| 3538-3546 | VTQNIINSL | 97.96 |
| 3645-3662 | PLFWYLVMSNSSYINLSK | 96.86 |
| 3721-3727 | NLNLFIN | 96.32 |
| 3739-3753 | KFQIWAFRYGFSTIA | 95.94 |
| 3820-3825 | QFVKFC | 95.32 |
| 3879-3884 | LIYAIN | 97.99 |

**Table S3. Aggregative sequences found in *Pf*UT using the WALTZ algorithm.** Colored sequences are ≤ 5 amino acids away from each other.

| **Residues** | **Aggregative Sequence** | **WALTZ score** |
| --- | --- | --- |
| 3-18 | KYLLFENSQYSYIINS | 95.21 |
| 116-122 | SFFKIIQ | 94.98 |
| 274-281 | TEIIINSE | 97.99 |
| 314-321 | KFFLSNIN | 96.57 |
| 323-346 | VDVKYITIIYTATCCIYTILDIYP | 95.40 |
| 357-363 | EAVYILN | 97.99 |
| 377-382 | VILKIF | 94.31 |
| 409-414 | YNVNIQ | 96.99 |
| 417-429 | IFFCIIQMINNIT | 95.86 |
| 441-452 | YCNIFINFFHYH | 97.07 |
| 455-461 | HILNIIC | 96.46 |
| 678-687 | IESIYNINIR | 97.99 |
| 702-721 | NLYAFMETFYIISILVHYSN | 97.16 |
| 748-763 | NNFLIIYILFTLYSFL | 96.20 |
| 787-796 | FYENIGEFIN | 92.64 |
| 901-907 | NIYYIYE | 97.52 |
| 917-936 | LCILICLSVFISLYKISLTC | 94.70 |
| 948-953 | YIFSYN | 98.33 |
| 986-992 | FTLLILK | 93.98 |
| 1023-1031 | GGLLFNITH | 97.73 |
| 1043-1048 | VLLHIV | 94.31 |
| 1076-1090 | KIYNIIFMYFYDIFN | 93.87 |
| 1108-1112 | KDLLF | 96.32 |
| 1122-1132 | VFLEYSELFFN | 97.08 |
| 1145-1151 | NICSYFK | 93.31 |
| 1182-1194 | ILCYYYLIYLFSN | 98.12 |
| 1202-1208 | SCIYFYQ | 95.13 |
| 1216-1226 | YFFLYQSLLYG | 99.33 |
| 1266-1277 | RLYIFLYALLFV | 97.94 |
| 1325-1345 | LVVKIFDSYHYYLIINNLSFK | 97.60 |
| 1387-1392 | YYDYIN | 92.98 |
| 1462-1469 | QDFSYNVY | 96.99 |
| 1647-1651 | KVVNI | 96.99 |
| 1730-1747 | EYNLYNYFNNNRYINYIP | 96.77 |
| 1752-1767 | YENLFNESINNNLTID | 96.99 |
| 1775-1780 | MYNNYN | 98.33 |
| 1796-1801 | NVSIFG | 93.98 |
| 1837-1845 | KQNNFNDYF | 97.66 |
| 1935-1944 | GSFNIFETFN | 96.49 |

**Table S4. Low complexity regions found in *Pf*UT using the Pfam algorithm**. Amino acid stretches overlapping with highlighted regions in Table S3 are indicated with the same color.

| **Type of domain** | **Residues** |
| --- | --- |
| low_complexity | 76-99 |
| low_complexity | 130-144 |
| low_complexity | 146-190 |
| low_complexity | 197-250 |
| low_complexity | 254-273 |
| low_complexity | 267-313 |
| low_complexity | 327-341 |
| low_complexity | 508-518 |
| low_complexity | 554-564 |
| low_complexity | 573-587 |
| low_complexity | 613-637 |
| low_complexity | 647-660 |
| low_complexity | 675-686 |
| low_complexity | 795-820 |
| low_complexity | 1043-1067 |
| low_complexity | 1397-1445 |
| low_complexity | 1474-1494 |
| low_complexity | 1504-1519 |
| low_complexity | 1544-1566 |
| low_complexity | 1567-1592 |
| low_complexity | 1610-1627 |
| low_complexity | 1660-1680 |
| low_complexity | 1697-1717 |
| low_complexity | 1731-1745 |
| low_complexity | 1768-1780 |
| low_complexity | 1890-1912 |
| low_complexity | 2041-2069 |
| low_complexity | 2091-2104 |
| low_complexity | 2118-2138 |
| low_complexity | 2244-2255 |
| low_complexity | 2300-2319 |
| low_complexity | 2652-2674 |
| low_complexity | 2683-2694 |
| low_complexity | 2694-2703 |
| low_complexity | 2878-2894 |
| low_complexity | 2990-3010 |
| low_complexity | 3009-3022 |
| low_complexity | 3036-3107 |
| low_complexity | 3113-3127 |
| low_complexity | 3138-3150 |
| low_complexity | 3155-3171 |
| low_complexity | 3187-3206 |
| low_complexity | 3213-3235 |
| low_complexity | 3406-3434 |
| low_complexity | 3514-3529 |
| Pfam | 3558-3893 |
| low_complexity | 3565-3579 |
| low_complexity | 3590-3607 |

**Table S5. Primer sequences used in this study.** In red are indicated homology arms. *Age*I restriction site is underlined. *Pac*I restriction site is double-underlined.

| **Primer** | **Sequence (5'-3')** | **Use** |
| --- | --- | --- |
| **P1** | TTCGAATAAAACCGGATGAAGGACTTATTATTCTAGATTAATCACCGTATC | Cloning of KDLLF into p*HHI-cambsd* |
| **P2** | AAGCTTATTTTGGCCTACTTCCTGAATAATAAGATCTAATTAGTGGCATAG | Cloning of KDLLF into p*HHI-cambsd* |
| **P3** | TTCGAATAAAACCGGATGAAAGTTGTGAACATATAGATTAATCACCGTATC | Cloning of KVNNI into p*HHI-cambsd* |
| **P4** | AAGCTTATTTTGGCCTACTTTCAACACTTGTATATCTAATTAGTGGCATAG | Cloning of KVNNI into p*HHI-cambsd* |
| **P5** | ACCGGTATGCTTATTCACGTAATAAAAAGTCC | Cloning of *Pf*UTf into p*HHI-cambsd* |
| **P6** | TTAATTAACTACTGGTCGTACTCCATCATCC | Cloning of *Pf*UTf into p*HHI-cambsd* |
| **P7** | GAAATATATCAGGATCCATGGCACC | Diagnostic PCR |
| **P8** | CACGTACGTGTACGTACATTTATCG | Diagnostic PCR |
| **P9** | TACCCTCGAGGGATCCATGCTTATTCACGTAATAAAAAGTCC | Cloning of *Pf*UTf into p*ColdTF* |
| **P10** | TAGACTGCAGGTCGACTCATGGTCGTACTCCATCATC | Cloning of *Pf*UTf into p*ColdTF* |

**
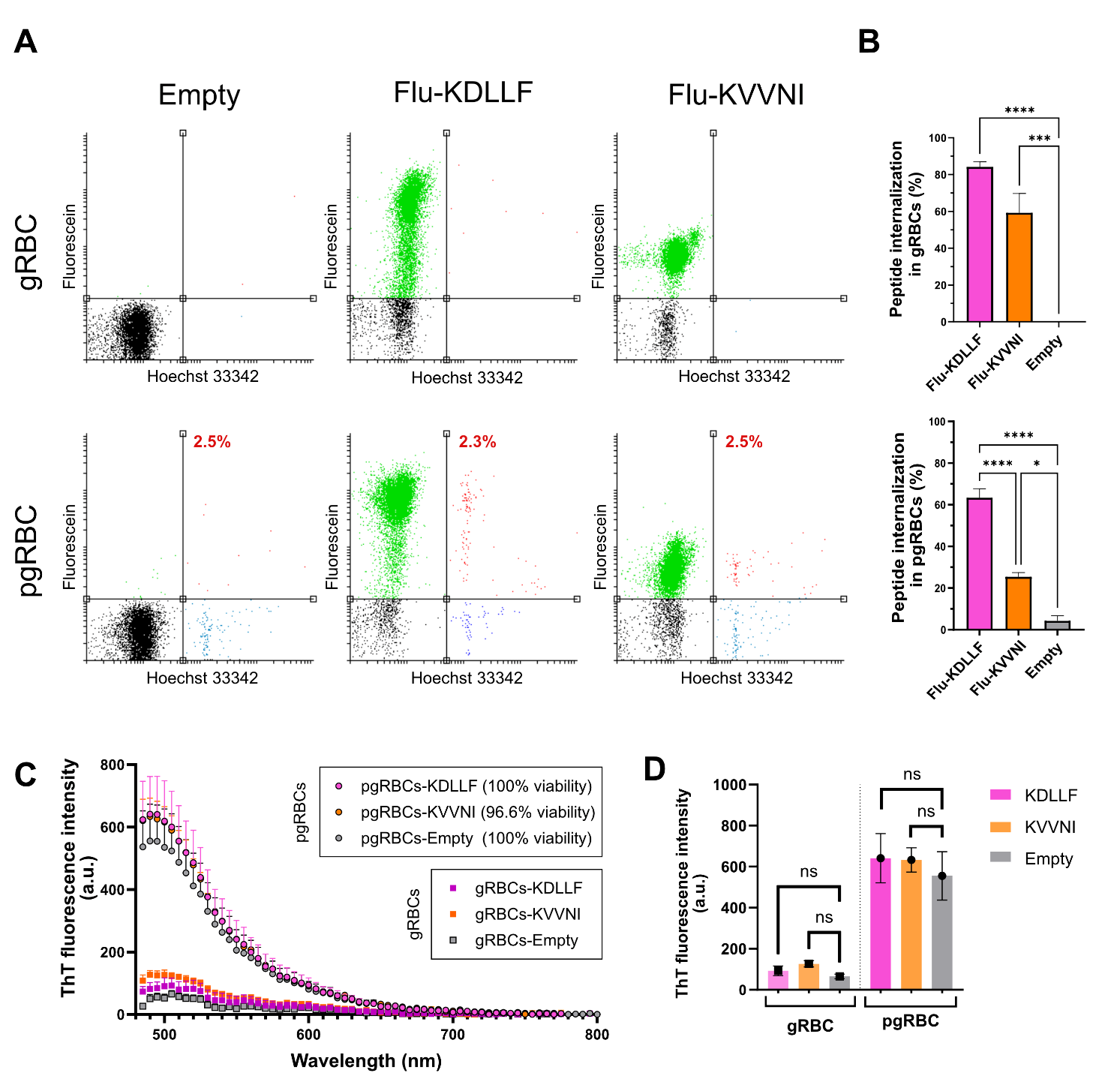
**

**Figure S1. Study of *P. falciparum* cultures grown in ghost RBCs loaded with the *Pf*UT-derived peptides KDLLF and KVVNI. (A)** Representative flow cytometry dot plots showing the fluorescein-labeled peptide internalization in ghost RBCs (gRBCs) and parasitized gRBCs (pgRBCs). Percentages in pgRBC panels indicate the parastiemias in the three samples. “Empty” refers to a gRBC or pgRBC preparation not loaded with either peptide. **(B)** Quantification of peptide internalization in gRBCs and pgRBCs. Bars represent the mean ± SEM of at least three independent experiments (10,000 events recorded). *: *p* < 0.05, ***: *p* < 0.001, ****: *p* < 0.0001. **(C)** ThT fluorescence aggregation analysis of peptide-loaded and non-loaded gRBC and pgRBC culture extracts, normalized to have equal protein content. Viability of the pgRBCs during the experiment is stated in the legend. Values represent mean ± SEM of at least three independent replicas. a.u.: arbitrary units. **(D)** Statistical analysis of the protein aggregation data in panel C (fluorescence intensity measured at the maximum ThT emission wavelength). Values represent mean ± SEM of at least three independent replicas. ns: not significant.

**
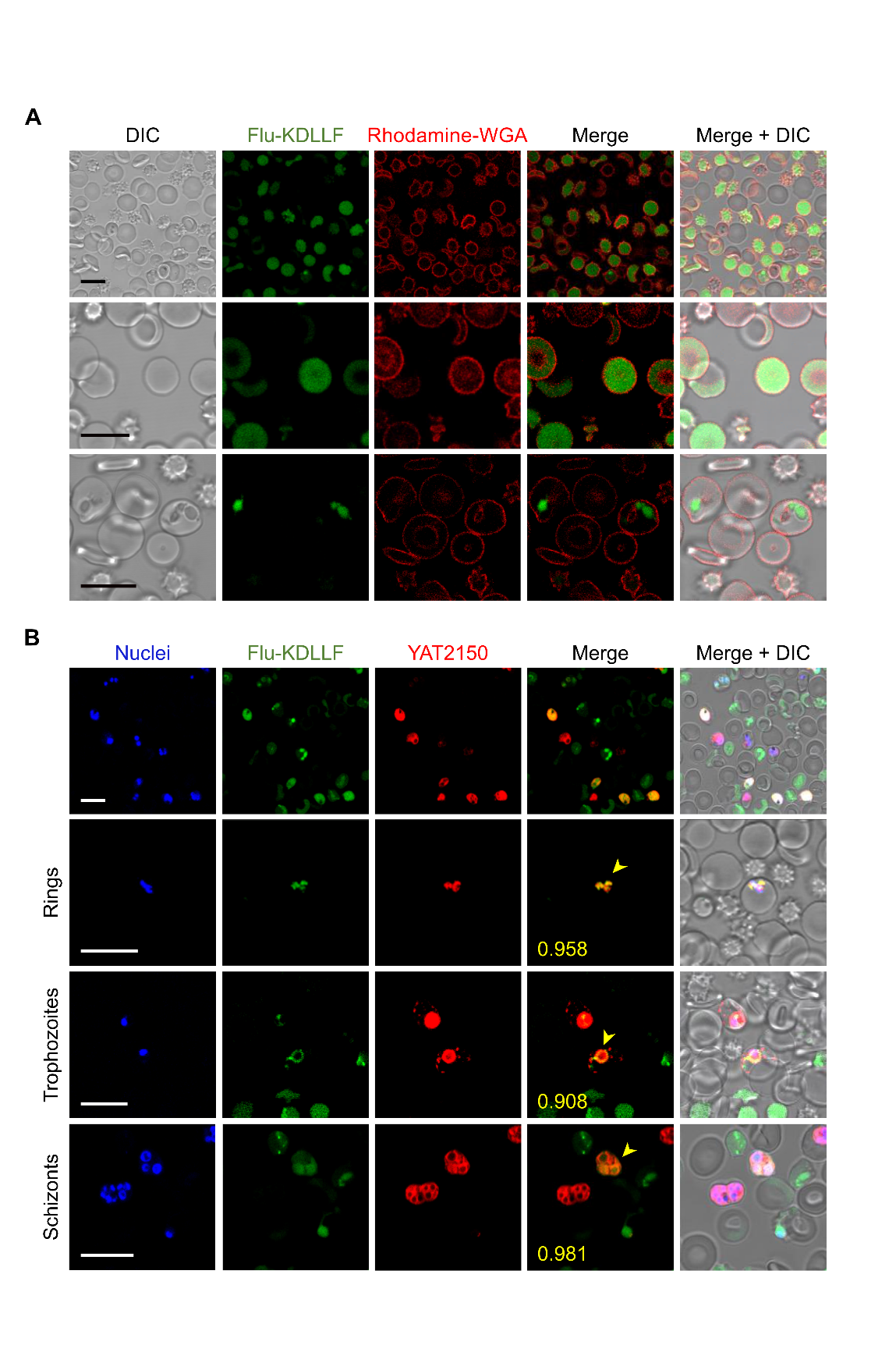
**

**Figure S2. Confocal fluorescence microscopy analysis of fluorescein-labeled KDLLF peptide in ghost RBCs.** **(A)** Representative confocal fluorescence microscopy images of peptide-loaded gRBCs, with membranes labeled with rhodamine-WGA. Higher magnification images in the lower rows show two distinct distribution patterns, with the peptide either having a homogenous distribution in the cell (second row) or forming condensate-like structures not surrounded by a membrane (third row). **(B)** Representative confocal fluorescence microscopy images of peptide-loaded gRBCs parasitized with *P. falciparum* 3D7. Cells were stained with YAT2150, to detect aggregated protein regions, and with Hoechst 33342 as a nuclear dye. Higher magnifications in the lower rows show cases of colocalization of the fluorescein-labeled peptide and YAT2150, with individual characteristic stages indicated by arrowheads. The corresponding Manders’ correlation coefficients are indicated in yellow digits. Scale bars = 10 µm; DIC: differential interference contrast.

**
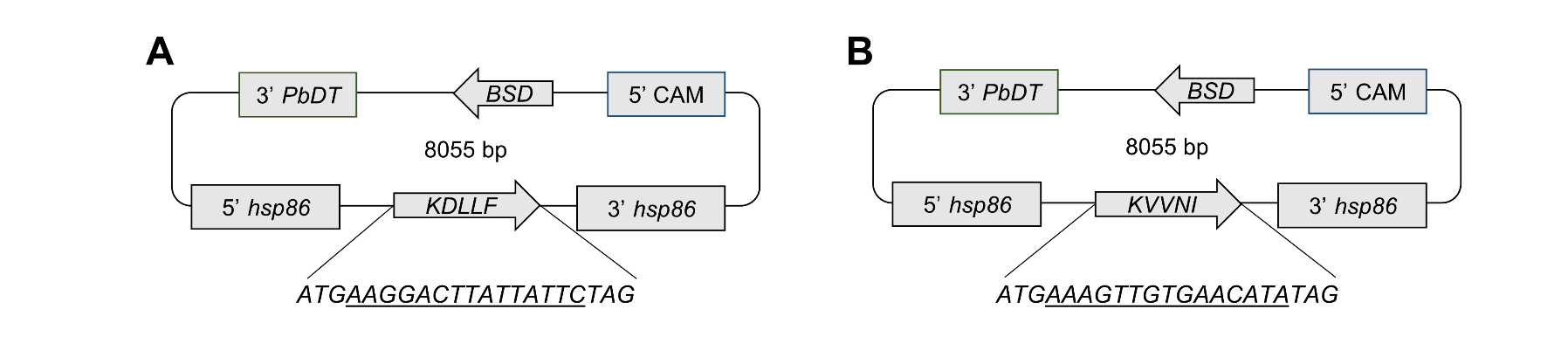
**

**Figure S3. Plasmid constructs for homologous seeding.** Map of the **(A)** p*HHI-cambsd_KDLLF*, and **(B)** p*HHI-cambsd_KVVNI* constructs, including start (ATG) and stop (TAG) codons. The peptide-encoding sequences are underlined.

**
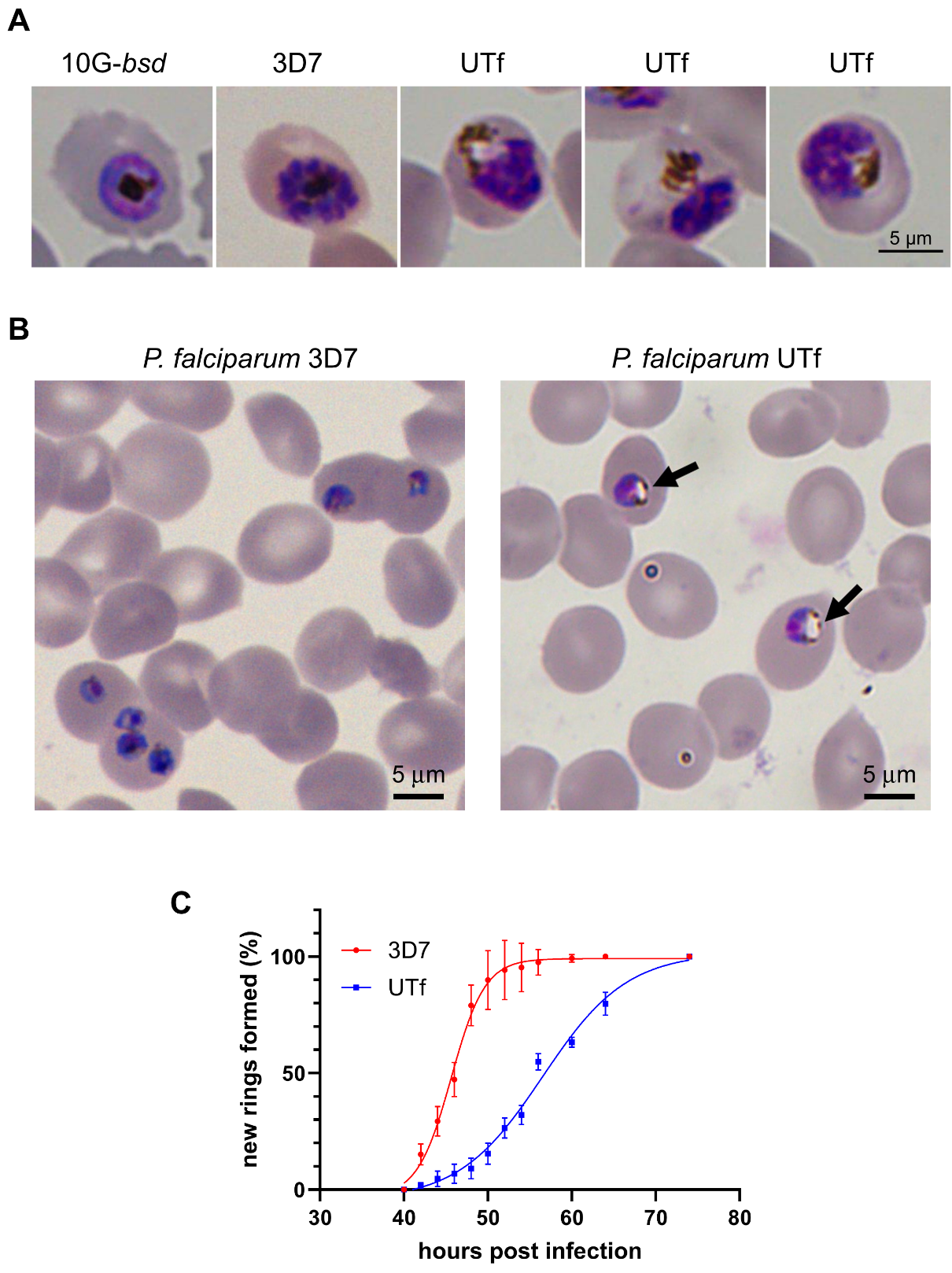
**

**Figure S4. Phenotypic alterations in *P. falciparum* UTf. (A,B)** Representative Giemsa-stained smears of *P. falciparum* strains 3D7, UTf, and 10G-*bsd* to show **(A)** the peripheral localization in UTf of hemozoin (brown areas) and **(B)** the presence of enlarged vesicles in the UTf strain. Arrows indicate the presence of hemozoin, which is formed during the trophozoite stage. **(C)** Asexual blood cycle duration in the UTf line compared with its parental 3D7 line. Percentages indicate the proportion of rings observed relative to the total number of rings at the end of the assay. The mean percentage of rings ± SD from five independent experiments is shown. Data was fitted to a sigmoidal curve with variable slope to extract the intraerythrocytic developmental cycle.

**
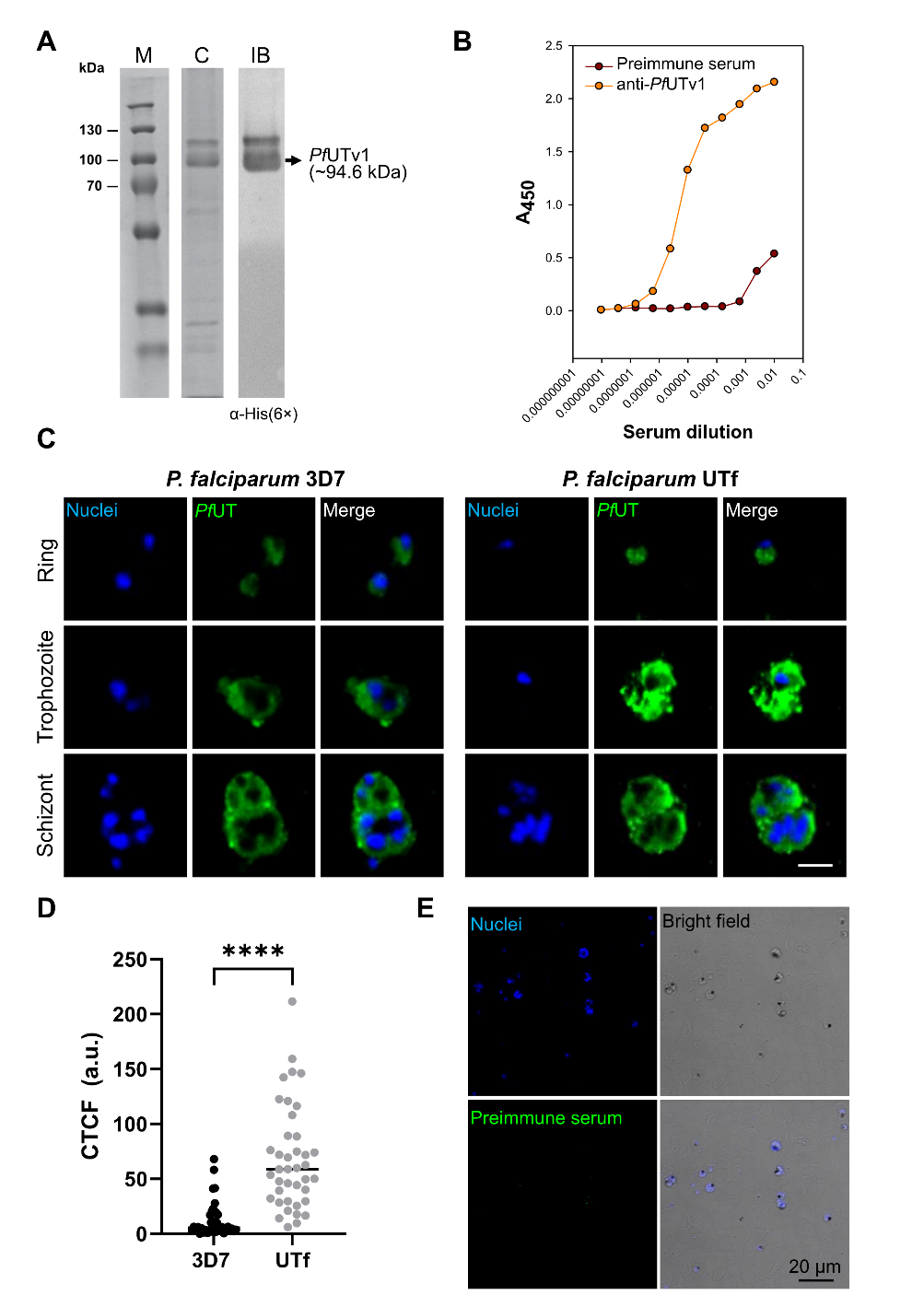
**

**Figure S5. Purification and validation of recombinant His-*Pf*UTv1. (A)** SDS-PAGE and immunoblot analysis of purified His-*Pf*E3ULv1 (*Pf*UTv1) to confirm the purity of the recombinant protein. The detected protein matched the expected molecular weight of 94.6 kDa (42.6 kDa from *Pf*UTv1 plus 52.0 kDa from the TF chaperone). Upper bands may represent aggregated forms of the protein. M: Molecular weight ladder. C: Coomassie Blue. IB: immunoblot with anti-His(6×). **(B)** Antibody specificity assessed by ELISA. Results show the binding specificity of raised antibodies to the recombinant protein compared to preimmune serum. **(C)** Representative images of indirect immunofluorescence assays in different stages of *P. falciparum* 3D7 and UTf strains. Rabbit polyclonal antibodies raised against *Pf*UTv1 were detected using anti-rabbit IgG Alexa 488-labeled secondary antibodies (green), and nuclei were counterstained with Hoechst 33342 (blue). Scale bar = 5 µm. **(D)** Corrected total cell fluorescence (CTCF) calculated from at least 40 different images of either the *P. falciparum* 3D7 or UTf lines. ****: *p* <0.0001. **(E)** Representative images of indirect immunofluorescence assays of the 3D7 strain probed with rabbit preimmune serum (green) as control. Nuclei were counterstained with Hoechst 33342 (blue).

**
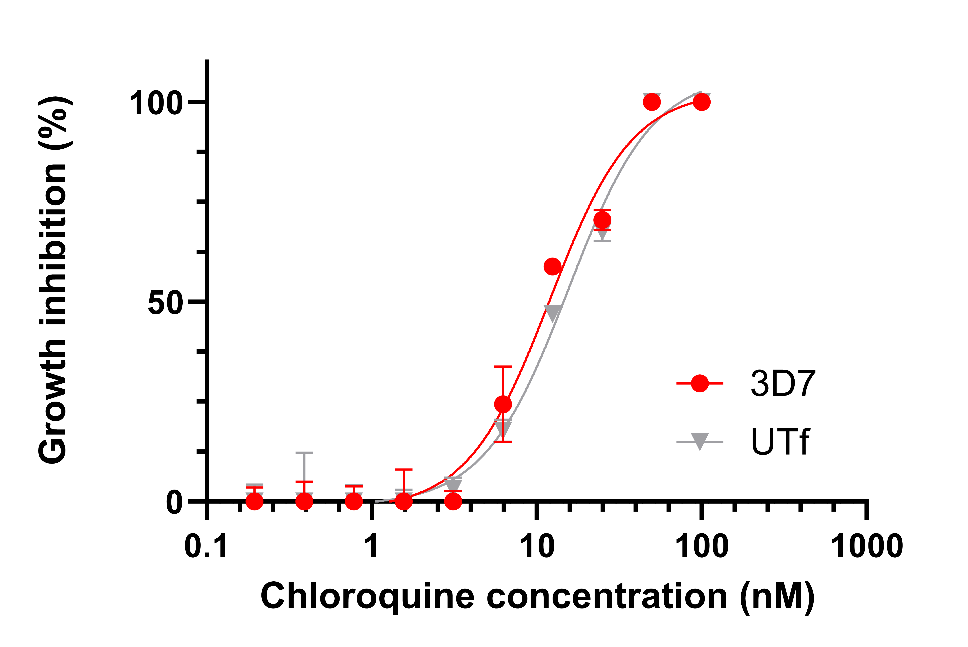
**

**Figure S6. Chloroquine growth inhibition assay in a *P. falciparum* culture.** Trophozoite-stage 3D7 and UTf parasites were treated with different concentrations of chloroquine (0.195-100 nM). The mean dose-dependent effect on parasite growth ± SD from three independent experiments is shown for each line.

**
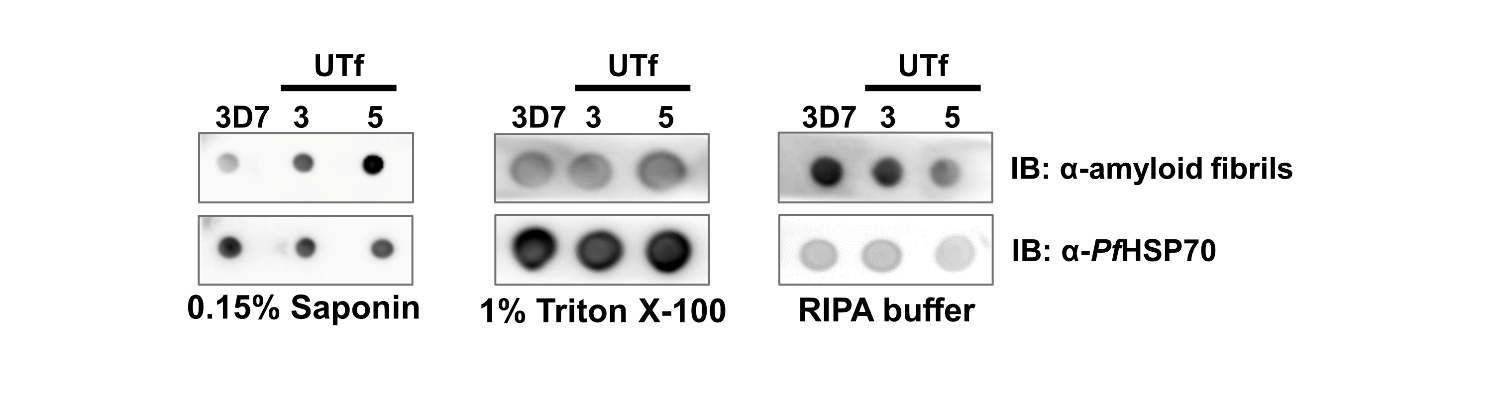
**

**Figure S7. Immunoblot detection of amyloid-like structures in *P. falciparum* 3D7 and UTf parasites.** Representative dot-blot analyses of different infected RBC-derived extracts of *P. falciparum* 3D7 and UTf strains, the latter maintained in the presence of either 3 µg/mL (3) or 5 µg/mL BS (5), probed with antibodies against amyloid fibrils and *Pf*HSP70. IB: antibodies used in the corresponding dot blots.
